# Phytochemicals Derived from Agricultural Residues and Their Valuable Properties and Applications

**DOI:** 10.3390/molecules28010342

**Published:** 2023-01-01

**Authors:** Marta Oleszek, Iwona Kowalska, Terenzio Bertuzzi, Wiesław Oleszek

**Affiliations:** 1 Department of Biochemistry and Crop Quality, Institute of Soil Science and Plant Cultivation, State Research Institute, 24-100 Puławy, Poland; 2 DIANA, Department of Animal Science, Food and Nutrition, Faculty of Agricultural, Food and Environmental Sciences, Università Cattolica del Sacro Cuore, Via E. Parmense, 84, 29122 Piacenza, Italy

**Keywords:** bioactive compounds, antioxidants, agricultural residues, fruits, vegetables, mass spectrometry, extraction

## Abstract

Billions of tons of agro-industrial residues are produced worldwide. This is associated with the risk of pollution as well as management and economic problems. Simultaneously, non-edible portions of many crops are rich in bioactive compounds with valuable properties. For this reason, developing various methods for utilizing agro-industrial residues as a source of high-value by-products is very important. The main objective of the paper is a review of the newest studies on biologically active compounds included in non-edible parts of crops with the highest amount of waste generated annually in the world. The review also provides the newest data on the chemical and biological properties, as well as the potential application of phytochemicals from such waste. The review shows that, in 2020, there were above 6 billion tonnes of residues only from the most popular crops. The greatest amount is generated during sugar, oil, and flour production. All described residues contain valuable phytochemicals that exhibit antioxidant, antimicrobial and very often anti-cancer activity. Many studies show interesting applications, mainly in pharmaceuticals and food production, but also in agriculture and wastewater remediation, as well as metal and steel industries.

## 1. Introduction

The agricultural industry generates billions of tonnes of waste from the tillage and processing of various crops. The crops with the largest amounts of produced residues are rice, maize, soybean, sugarcane, potato, tomato, and cucumber, as well as some fruits, mainly bananas, oranges, grapes, and apples [1,2]. It has been estimated that European food processing companies generate annually approximately 100 Mt of waste and by-products, mostly during the production of drinks (26%), dairy and ice cream (21.3%), and fruits and vegetables (14.8%) [3].

In Table 1, the amounts of particular wastes generated worldwide are presented. Many of them are rich in biologically active compounds and have the potential to become important raw materials for obtaining valuable phytochemicals. Vegetable and fruit processing by-products are promising sources of valuable phytochemicals having antioxidant, antimicrobial, anti-inflammatory, anti-cancer, and cardiovascular protection activities [4]. The applications of these agro-industrial residues and their bioactive compounds in functional food and cosmetics production were presented in many studies [5,6,7]. Moreover, due to the potential health risk of some synthetic antioxidants such as BHA, the identification and isolation of natural antioxidants from waste has become increasingly attractive. Important criteria to decide if a product or by-product can be of interest to recover phytochemicals are the absolute concentration and preconcentration factor, as well as the total amount of product or by-product per batch [8].

**Figure 1:**
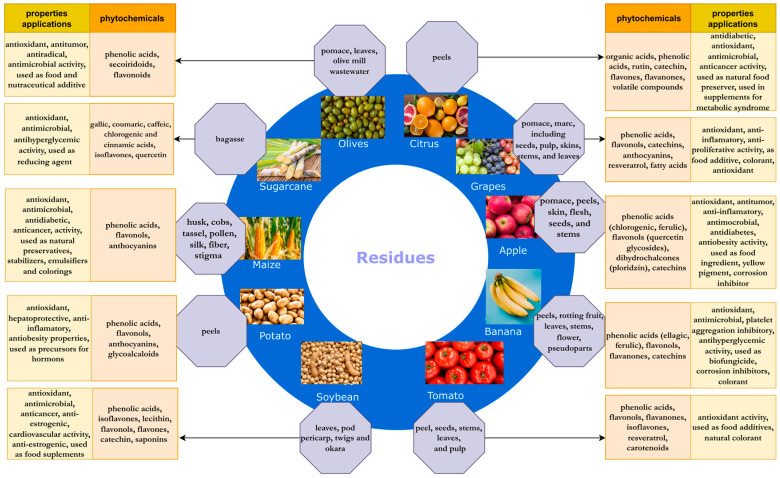
Agricultural residues and the properties and applications of their phytochemicals.

As interest in waste processing has been growing in recent years, many scientific papers have been published on new compounds in agro-industrial waste, new properties of valuable phytochemicals contained in crop residues and their applications. It seems necessary to summarize and collect the latest knowledge on this subject. In this work, an overview of the recent knowledge on the phytochemicals in some of the most popular food by-products, with the highest amount generated in the world, as well as on their properties and potential applications, have been presented in more detail (Figure 1).

## 2. Phytochemicals from Crop Residues

### 2.1. Sugarcane Bagasse

Large amounts of waste are generated during the processing of sugarcane. In fact, one metric ton of sugarcane generates 280 kg of bagasse. Sugarcane bagasse is one of the most abundant agro-food by-products and is a very promising raw material available at low cost for recovering bioactive substances [18,19]. Sugarcane bagasse consists mainly of cellulose (35–50%), hemicellulose (26–41%), lignin (11–25%), but also some amount of plant secondary metabolites (PSM), mainly anthocyanins and mineral substances [20,21,22,23,24,25].

Phenolic compounds are a very important group of natural substances identified in sugarcane waste. Nonetheless, steam explosion and ultrasound-assisted extraction (UAE) pretreatment was applied for the production of valuable phenolic compounds from the lignin included in this residue. Chromatographic analysis revealed that sugarcane bagasse is a good feedstock for the generation of phenolic acids. The concentration of total phenolics with the Folin-Ciocalteau method was between 2.8 and 3.2 g/L. Zhao et al. [26] have identified many phenolics, mainly flavonoids and phenolic acids, in sugarcane bagasse extract (Table 2). The total polyphenol content was detected as higher than 4 mg/g of dry bagasse, with total flavonoid content of 470 mg quercetin/g of polyphenol. The most abundant phenolic acids identified in the sugarcane bagasse extract were gallic acid (4.36 mg/g extract), ferulic acid (1.87 mg/g extract) and coumaric acid (1.66 mg/g extract). Spectroscopic analysis showed that a predominant amount of *p*-coumaric acid is ester-linked to the cell wall components, mainly to lignin. On the other hand, about half of the ferulic acid is esterified to the cell wall hemicelluloses. The purified sugarcane bagasse hydrolysate consisted mainly of *p*-coumaric acid. Besides, the purified products showed the same antioxidant activity, reducing power and free radical scavenging capacity as the standard *p*-coumaric acid. Al Arni et al. [27] stated that the major natural products contained in the lignin fraction were *p*-coumaric acid, ferulic acid, syringic acid, and vanillin.

Gallic, coumaric, caffeic, chlorogenic, and cinnamic acids were the main phenolic compounds extracted from raw and alkaline pretreated sugarcane bagasse and identified by high-performance liquid chromatography (HPLC) [28]. The aromatic phenolic compounds (*p*-coumaric acid, ferulic acid, *p*-hydroxybenzaldehyde, vanillin, and vanillic acid) were reported in sugarcane bagasse pith. Five phenolic compounds (tricin 4-*O*-guaiacylglyceryl ether-7-*O*-glucopyranoside, genistin, *p*-coumaric acid, quercetin, and genistein) in 30% hydroalcoholic fraction of sugarcane bagasse were identified using ultra-high performance liquid chromatography/high-resolution time of flight mass spectrometry (UHPLC-HR-TOF-MS); (Table 2). The total phenolic content was 170.68 mg gallic acid/g dry extract [19].

Phenolic compounds derived from sugarcane bagasse exhibited many biological activities, which were used in various applications. The most important biological activities and the newest and most interesting applications have been summarized in Table 3.

### 2.2. Maize Residues

Maize (corn *Zea mays* L.) bran, husk, cobs, tassel, pollen, silk, and fiber are residues of corn production. They contain substantial amounts of phytochemicals, such as phenolic compounds, carotenoid pigments and phytosterols [39] (Table 4). 

Corn bran is produced as a plentiful by-product during the corn dry milling process. Similar to other cereal grains, phenolics in corn bran exist in free insoluble bound and soluble-conjugated forms. Corn bran is a rich source of ferulic acid compared to other cereals, fruits and vegetables. Guo et al. [39] isolated four forms of ferulic acid and its derivates from corn bran. On the other hand, it has been reported that the hexane-derived extract from corn bran contains high levels of ferulate-phytosterol esters, similar in composition and function to oryzanol.

Another corn waste is a husk. It is the outer leafy covering of an ear of *Zea mays* L. The main constituents of the maize husk extracts determined in various phytochemical studies are phenolic compounds, e.g., flavonoids [41,50]. Saponins, glycosides, and alkaloids are present mainly in the aqueous and methanolic extracts, while phenols and tannins are numerous in methanolic ones [51]. Moreover, corn husk has high contents of anthocyanins [48,52]. Simla et al. [53] reported that anthocyanins concentration in corn husks ranges from 0.003 to 4.9 mg/g. The major anthocyanins of corn husk were identified as malonylation products of cyanidin, pelargonidin, and peonidin derivatives [54].

Important by-products of the corn industry are cobs. For every 100 kg of corn grain, approximately 18 kg of corn cobs are produced. Corn cob is one of the food waste-material having a phytochemical component that has a healthy benefit [55]. They contain cyanidin-3-glucoside and cyanidin-3-(6″malonylglucoside) as main anthocyanins, as well as pelargonidin-3-glucoside, peonidin-3-glucoside and their malonyl counterparts [48].

Corn tassel is a by-product from hybrid corn seed production and an excellent source of phytochemicals (the flavonol glycosides of quercetin, isorhamnetin and kaempferol) with beneficial properties [56]. In Thailand, purple waxy corn is considered a special corn type because it is rich in phenolics, anthocyanins, and carotenoids in the tassel [57]. Besides, corn tassels could be considered a great source of valuable products such as volatile oils.

Corn pollen is another corn waste. Significant amounts of phytochemicals, including carotenoids, steroids, terpenes and flavonoids, are present in maize pollen [52]. Bujang et al. (2021) showed that maize pollen contains a high total phenolic content and total flavonoid content of 783.02 mg gallic acid equivalent (GAE)/100 g and 1706.83 mg quercetin equivalent (QE)/100 g, respectively. The flavonoid pattern of maize pollen is characterized by an accumulation of the predominant flavonols, quercetin and traces of isorhamnetin diglycosides and rutin. According to Žilić et al. [58], the quercetin values in maize pollen were 324.16 μg/g and 81.61 to 466.82 μg/g, respectively.

Corn silk, another by-product from corn processing, contains a wide range of bioactive compounds in the form of volatile oils, steroids, saponins, anthocyanins [59], and other natural antioxidants, such as flavonoids [52] and phenolic compounds [41,58,59]. In the corn silk powder, the high phenolic content (94.10 ± 0.26 mg GAE/g) and flavonoid content (163.93 ± 0.83 mg QE/100 g) are responsible for its high antioxidant activity [60]. About 29 flavonoids have been isolated from corn silk. Most of them are C-glycoside compounds and have the same parent nucleus as luteolin [44]. Ren et al. [61] successfully isolated and separated compounds such as 2″-*O*-*α*-l-rhamnosyl-6-C-3″-deoxyglucosyl-3′-methoxyluteolin, ax-5′-methane-3′-methoxymaysin, ax-4″-OH-3′-methoxymaysin, 6,4′-dihydroxy-3′-methoxyflavone-7-*O*-glucoside, and 7,4′-dihydroxy-3′-methoxyflavone-2″-*O*-*α*-l-rhamnosyl-6-C fucoside from corn silk. Moreover, among flavonoids, Haslina and Eva [43] determined in corn silk: apigmaysin, maysin, isoorientin-2″-*O*-*α*-l-rhamnoside, 3-methoxymaysine, and ax-4-OH maysin. 

This richness of biologically active compounds results in advantageous properties and applications. The most important properties and the newest studies on the application are listed in Table 5.

### 2.3. Potato Waste

Approximately 40–50% of potatoes are not suitable for human consumption. Industrial processing of potatoes (mashed and canned potatoes, chips, fries and ready meals) creates huge amounts of peel as waste [66,67]. Potato peel is a non-edible residue generated in considerable amounts by food processing plants. Depending on the peeling process, e.g., abrasion, lye or steam peeling, the amount of waste can range between 15 and 40% of the number of processed potatoes [68]. Industrial processing produces between 70 to 140 thousand tons of peels worldwide annually, which are available to be used in other applications [69].

Potato peels differ greatly from other agricultural by-products because they are revalorized as a source of functional and bioactive compounds, including phenolic compounds, glycoalkaloids, vitamins and minerals [70] (Table 6).

Potato peel is a good source of phenolic compounds because almost 50% of potato phenolics are located in the peel and adjoining tissues [74,83]. The results obtained by Wu et al. [77] showed that the potato peels contained a higher amount of phenolics than the flesh. Moreover, the polyphenols in potato peel are ten times higher than those in the pulp. Potato peel extract contains 70.82 mg of catechin equivalent (CE)/100 g of phenolic and had a high level of phenolic compounds (2.91 mg GAE/g dry weight) that was found to be greater than carrot (1.52 mg GAE/g dry weight), wheat bran (1.0 mg GAE/g dry weight), and onion (2.5 mg GAE/g dry weight) [67]. The results of Javed et al. [72] showed that the total phenolic content in potato peel ranged from 1.02 to 2.92 g/100 g and total flavonoids ranged from 0.51 to 0.96 g/100 g. Phenolic acids are the most abundant phenolic compounds in potato peel. They include derivatives of hydroxycinnamic and hydroxybenzoic acids (Table 6). Kumari et al. [84], using UHPLC-MS/MS, showed that chlorogenic and caffeic acids are important components of the free-form phenolics in potato peel. The results show that phenolic acids in potato peals are not only present in their free form but also occur in bound form. Javed et al. [72] showed that the extract of potato peel contains chlorogenic acid (753.0–821.3 mg/100 g), caffeic acid (278.0–296.0 mg/100 g), protocatechuic acid (216.0–256.0 mg/100 g), *p*-hydroxybenzoic acid (82.0–87.0 mg/100 g), gallic acid (58.6–63.0 mg/100 g), vanillic acid (43.0–48.0 mg/100 g), and *p*-coumaric acid (41.8–45.6 mg/100 g). Silva–Beltran et al. [78] showed that flavonoids such as rutin and quercetin were present in potato peel at low concentrations of 5.01 and 11.22 mg/100 g dry weight, respectively.

Many studies have noted that potato peels are excellent untapped source of steroidal alkaloids, e.g., glycoalkaloids (*α*-solanine and *α*-chaconine) and aglycone alkaloids (solanidine and demissidine; Table 6) [80,81,85]. *α*-solanine, *α*-chaconine, and the glycosides of solanidine constitute about 95% of the total potato peel glycoalkaloid content [86]. Higher amounts of these compounds were found in potato peel, unlike potato flesh [87]. There are various cultural, genetic and storage factors that influence the concentration of glycoalkaloids in potato peel [88]. Concerning cultivars, it was shown that the variety with blue flesh showed the highest concentration (5.68 mg/100 g fresh weight), followed by the red-leaved (5.26 mg/100 g fresh weight), while yellow or cream flesh. In the study of Singh et al. [89] of potato peel, glycoalkaloids were detected as 1.05 mg/100 g. The results of Rytel et al. [88] showed that the glycoalkaloid content of potato peel depends on the potato cultivar and ranges from 181 mg/kg to 3526 mg/kg of fresh potato tubers.

Besides, the peel of pigmented potatoes is an excellent source of anthocyanins, e.g., pelargonidin-3-(*p*-coumaryoly rutinoside)-5-glucoside and petunidin-3-(*p*-coumaroyl rutinoside)-5-glucoside. It has been proven that their content depends on the cultivar [90]. Ji et al. [80] showed that anthocyanidin levels were higher in the peel than in the tuber. The most important beneficial properties and potential applications of phytochemicals identified in potato waste are listed in Table 7.

### 2.4. Soybean Residues

Soybean waste has the potential as a sustainable source of phytochemicals and functional foods. It includes both leaves, pod pericarp, and twigs, as well as the residues after seeds processing, so-called okara. Okara is the residue of soybean milling after extraction of the aqueous fraction used for producing tofu and soy drink and presents high nutritional value [109]. The results of the last studies showed that an okara contains enough bioactive compounds that make it useful to obtain value-added products for use in food production, oil extraction, nutraceutical, pharmaceutical, and cosmetic formulations. Moreover, it was stated that okara isoflavones have good antioxidant activity. Although some nutrients like protein decrease in okara during soymilk processing, it still has many other phytochemicals and nutrients, making it their least expensive and most excellent source. Since it has good antimicrobial activity, it can be used in pharmaceutical industries, thus opening up new frontiers for drug exploration [109]. Various food enriched with okara, such as biscuits and cookies, have been mentioned in the literature [110,111]. Guimarăes et al. [112] reported that food products enriched with okara contained 0.411 mg/100 mL of *β*-carotene and 0.15 μm/g isoflavones.

One of the main phytochemicals in soybean waste are isoflavones: daidzein, genistein, glycitein, and their glycosides (e.g., acetyl-, malonyl-, and *β*-glycosides) [113]. Isoflavones are compounds belonging to the flavonoid group. In addition to the well-established antioxidant effect, isoflavones exhibit estrogenic activity because of their similar structure to estrogen [113,114]. The beneficial effects of isoflavones are the prevention of hormone-dependent cancer, coronary heart disease, osteoporosis, and menopausal symptoms [114]. Kumar et al. [115] proved that daidzein expressed anticancer activity against human breast cancer cells MCF-7. The extract from soybean waste material showed total phenolic content (TPC) in the range of 27.4–167 mg GAE/g, total flavonoids from 10.4 to 63.8 mg QE/g and antioxidant activity (AOA) from 26.5% to 84.7% [114]. Moreover, their values were highest in the leaves, followed by pod pericarp and twigs. As was stated by Šibul et al. [113], soybean roots are also a good source of daidzein and genistein, as well as other phenolic compounds. The concentrations of isoflavones in roots were higher than in herbs, 1584.5 and 93.48 μg/g of dry extract, respectively. The newest study on soybean pods stated that its ethanolic extract and fractions exhibited anticancer potential against human colorectal carcinoma (HTC-116) and prostate cancer (PC-3) [116]. Moreover, it was the first analysis of this material using ultra-*high*-*performance liquid chromatography* coupled with electrospray ionization quadrupole time-of-flight mass spectrometry (UPLC-ESI-QTOF-MS), resulting in the identification of 50 polyphenols belonging to phenolic acids, flavonoids and other groups. The authors stated that soybean pods might be useful material as an active food additive or a component in dietary supplements and preparations with anti-radical and anti-cancer properties.

Soybean by-products are a good source of lecithin. Lecithin is a natural emulsifier that stabilizes fat and improves the texture of many food products, such as salad dressings, desserts, margarine, chocolate, and baking and cooking goods [117]. Moreover, it also has health benefits such as lowering cholesterol and low-density lipoprotein level in the human blood, improving digestion, cognitive and immune function, as well as aiding in the prevention of gall bladder and liver diseases.

Saponins are another important group of phytochemicals derived from soybean waste [113]. Soyasaponins have been linked to anti-obesity, antioxidative stress, and anti-inflammatory properties, as well as preventive effects on hepatic triacylglycerol accumulation [118]. One of the latest applications of saponins derived from soybean by-products was as eco-friendly agents for washing pesticide residues in the vegetable and fruit industries [119].

Compounds identified and quantified in soybean waste are specified in Table 8. The newest studies on the applications and properties of soybean waste are presented in Table 9.

### 2.5. Tomato Residues

During the industrial processing of tomatoes, a considerable amount of waste is generated. Tomato waste consists mainly of peel, seeds, stems, leaves, fibrous parts and pulp residues [124]. The wet tomato pomace constitutes the major part of this waste, which consists of 33% seed, 27% peel and 40% pulp, while the dried pomace contains 44% seed and 56% pulp and peel [125]. When tomatoes are processed into products like ketchup, juice or sauces, 3–7% of their weight becomes waste. The management of tomato by-products is considered an important problem faced by tomato processing companies due to their disposal into the environment [126,127].

Although tomato waste has no commercial value, it is a rich source of nutrients, colorants and highly biologically active compounds such as polyphenols, carotenes, sterols, tocopherols, terpenes, and others (Table 10) [128,129,130,131,132]. The number of these compounds depends on tomato variety, part of the tomato residues (seed, peels, and pulp), time and extraction method, used solvent, as well as fractions gained after the isolation procedure, e.g., alkaline-hydrolyzable, acid-hydrolyzable, and bound phenolics [133]. They reported a total phenolics average of 1229.5 mg GAE/kg, of which flavonoids accounted for 415.3 mg QE/kg. The most abundant phenolic acids quantified in dried tomato waste were ellagic (143.4 mg/kg) and chlorogenic (76.3 mg/kg) acids. Other phenolic acids determined in lower concentrations were gallic, salicylic, coumaric, vanillic and syringic [133]. The levels of vanillic (26.9 mg/kg) and gallic (17.1 mg/kg) was lower than those found by Elbadrawy and Sello [134] in tomato peel (33.1 and 38.5 mg/kg, respectively). Ćetković et al. [135] identified phenolic acids (chlorogenic, *p*-coumaric, ferulic, caffeic and rosmarinic acid), flavonols (quercetin and rutin and its derivatives), and flavanone (naringenin derivatives) as the major phenolic compounds in extracts of tomato waste. The results obtained by Aires et al. [136] showed that the major polyphenol found in tomato wastes were kaempferol-3-*O*-rutinoside and caffeic acid. Several papers [135,136,137,138] reported the amounts of caffeic, chlorogenic, *p*-coumaric acids, kaempferol and quercetin, among other phenolic compounds found in tomato by-products. In the tomato’s wastes, Di Donato et al. [139] identified two main flavonoid compunds e.g., kaempferol rutinoside and quercetin rutinoside. Rutin and chlorogenic acid were the most abundant individual phenolics found by García–Valverde et al. [140] in all studied tomato varieties. 

Traditionally, the bioactivity of tomatoes and their products has been attributed to carotenoids (*β*-carotene and lycopene). The results of Nour et al. [133] confirmed that dried tomato wastes contain considerable amounts of lycopene (510.6 mg/kg) and *β*-carotene (95.6 mg/kg) and exhibited good antioxidant properties. The results obtained by Fărcaş et al. [145] confirmed lycopene as the main carotenoid of tomato waste in a concentration between 42.18 and 70.03 mg/100 g DW (dry weight). Simultaneously, peels contain around 5 times more lycopene compared to tomato pulp [146,147]. The lycopene content in peel was 734 μg/g DW, but significant amounts of *β*-carotene, cis-*β*-carotene and lutein were also determined. The study by Górecka et al. [148] showed that tomato waste could be considered a promising source of lycopene for the production of functional foods.

Peels, as one of the main residues of tomato, are a richer source of nutrients and biologically active compounds than the pulp [137,149]. Despite of high concentration of carotenoids, peels also contain a considerable amount of polyphenols. The results obtained by Hsieh et al. [97] showed that the main flavonoids detected in fresh tomato peel were quercetin, myricetin, apigenin, catechin, puerarin, fisetin, hesperidin, naringin, rutin and their levels were reported as 4.2, 2.9, 1.9, 0.9, 0.8, 0.5, 0.3, 0.2, and 0.2 mg/100 g, respectively. It has been proven that tomato peel extracts contain high amounts of kaemferol-3-*O*-rutinoside (from 8.5 to 142.5 mg/kg) [127], quercetin derivatives, *p*-coumaric acid and chlorogenic acid derivative [150,151]. The main phenolic acids identified in tomato peel are protocatechuic, vanillic, gallic, catechin and caffeic acid. Their corresponding concentrations were 5.52, 3.85, 3.31, 2.98, and 0.50 mg/100 g, respectively [134]. The results of Lucera et al. [152] showed that tomato peels contain 4.90 mg/g DW of total phenolic and 2.21 mg/g DW of total flavonoids. The total polyphenolic content in tomato peels and seeds was higher than in the pulp. On the other hand, tomato peel has a very small amount of anthocyanin [153].

Tomato seeds are considered a potential natural source of antioxidants due to their rich phytochemical profile. Many publications indicate that tomato seeds contain, e.g., carotenoids, proteins, polyphenols, phytosterols, minerals and vitamin E [154]. According to Eller et al. [155], the total content of phenolic compounds in the tomato seed extract was 20.66 mg/100 g. Quercetin-3-*O*-sophoroside, isorhamnetin-3-*O*-sophoroside, and kaempferol-3-*O*-sophoroside were present in the highest concentrations of the total phenolic compounds. Quercetin derivatives contributed approximately 37% of the total flavonoid content. Pellicanò et al. [156] found naringenin (84.04 mg/kg DW) as the most abundant flavonoid identified, followed by caffeic acid (26.60 mg/kg DW). Apart from phenolics, carotenoids are the next class of bioactive compounds present in tomato seeds. Qualitatively, the carotenoid composition (*β*-carotene and lycopene isoforms: lycopene all *trans*, lycopene *cis* 1, lycopene *cis* 2, lycopene *cis* 3) in tomato seeds is similar to that of the carotenoids in tomato fruit [157].

Tomato waste has attracted great interest due to its biological activity and potential applications of phytochemicals (Table 11).

### 2.6. Banana Residues

Banana (*Musa* spp., Musaceae family) is one of the main fruit crops cultivated for its edible fruits in tropical and subtropical regions. The main by-product of bananas is its peels, which represent approx. 30% of the whole fruit [164]. Moreover, banana waste also includes small-sized, damaged, or rotting fruit, leaves, stems, and pseudoparts. Banana peels are sometimes used as feedstock for livestock, goats, monkeys, poultry, rabbits, fish, zebras, and many other species. They are rich in vitamin B6, manganese, vitamin C, fiber, potassium, biotin, and copper [165], but also in phytochemicals with high antioxidant capacity such as phenolics (flavonols, hydroxycinnamic acids, gallocatechin), anthocyanin (delphinidin, cyanidin), carotenoids (*β*-carotenoids, *α*-carotenoids, and xanthophylls), catecholamines, sterols and triterpenes (Table 12). Banana peels are natural antacids and are helpful in acid reflux, heartburn, and diarrhea [165].

Previous studies reported that the banana peel is rich in chemical compounds as antioxidant and antimicrobial activities [167,168,169,171]. Moreover, ethanoic extract from banana peel exhibited the strongest antihyperglycemic activity in comparison with the extract from pulp, seed, and flower [172]. Phytochemicals derived from banana peel were tested as a biofungicide against *Fusarium culmorum* and *Rhizoctonia solani* and as a bactericide against *Agrobacterium tumefaciens* for the natural preservation of wood during handling or in service. Encapsulation is successfully investigated as the method for stabilizing the banana peel extract and its bioactive compounds during storage [173].

Other phytochemical components present in the banana peel extracts, such as ethanediol and butanediol, were determined as highly reducing agents to synthesize silver nanoparticles, which are significant to the medical and chemical industries [173].

The harvesting of the fruits in the plantation requires the decapitation of the whole; therefore, the valuable banana by-products, in addition to peels, are the pseudostem, leaves, inflorescence, and fruit stalk, but also rhizome, which can also be used as a raw material for the acquisition of phytochemicals [174]. Kandasamy et al. [170] isolated three compounds from the pseudostem and rhizome of bananas, including chlorogenic acids, cycloeucalenol acetate, and 4-epicyclomusalenone. Crude extract and isolated compounds are characterized by strong antibacterial, antifungal, antiplatelet aggregation, and anticancer activities.

Using the inflorescence of bananas, anthocyanins can be obtained as good biocolorants with attractive colors, moderate stability in food systems, water solubility, and benefits for health [175]. Cyanidin-3-rutinoside, as the main compound, could be exploited as a cheap source of natural food colorant. 

The newest application and explored properties of biologically active compounds from banana residues are presented in Table 13.

### 2.7. Apple Residues

Poland is the main producer of apples in the world, with an annual production of over 4 million tons [177]. About 25% of apple biomass was wasted during crop and processing. Apple pomace as a waste from apple juice and cider processing consists mainly of apple skin/flesh, seeds, and stems [178]. Until recently, apple waste was used as livestock feed, bioenergy feedstock, as well as for food supplementation and pectin extraction, but still, it is far from being used at its full potential, particularly considering its application in the pharmaceuticals and cosmetics industry [179,180]. Nonetheless, apple pomace has the potential to become a source of valuable biomaterials for agriculture. It contains numerous phytochemicals in the form of pectin and dietary fibers, but also polyphenols, triterpenoids, and volatiles. Interestingly, apple pomace is a richer source of antioxidants than fresh fruits itself because it has a significantly lower content of water; moreover, many valuable bioactive compounds are found mainly in the peels and seeds [180].

Polyphenols are the main valuable constituents of apple pomace. Waldbauer et al. [181] reported that the total phenolic content in apple pomace is in the range of 262–856 mg of total phenols/100 g. This content differs between studies due to the use of different solvents, extraction conditions, and apple varieties [182,183].

Four major phenolic groups are hydroxycinnamic acids, dihydrochalcone derivatives (phloretin and its glycosides), flavan-3-ols (catechin and procyanidins), and flavonols (quercetin and its glycosides) [184,185].

Although the phytochemical composition of apple pomace has been studied for a long time, new compounds with beneficial properties are still being isolated and identified. Ramirez-Ambrosi et al. [186] identified 52 phenolic compounds using a newly developed, rapid, selective, and sensitive strategy of ultrahigh-performance liquid chromatography with diode array detection coupled to electrospray ionization and quadrupole time-of-flight mass spectrometry (UHPLC-DAD–ESI-Q-ToF-MS) with automatic and simultaneous acquisition of exact mass at high and low collision energy. Among new compounds, two dihydrochalcones (two isomers of phloretin-pentosyl-hexosides) and three flavonols (isorhamnetin-3-*O*-rutinoside, isorhamnetin-3-*O*-pentosides and isorhamnetin-3-*O*-arabinofuranoside) have been tentatively identified for the first time in apple pomace.

One of the compounds newly identified in the last few years in apple pomace is monoterpene–pinnatifidanoside D [185]. This compound has been isolated for the first time from *Crataegus pinnatifida* and exhibited small antiplatelet aggregation activity.

Mohammed and Mustafa [187] and Khalil and Mustafa [188] isolated and structurally elucidated novel furanocoumarins from apple seeds. Isolated compounds exhibited promising antimicrobial activity against *Pseudomonas aeruginosa*, *Klebsiella pneumonia*, *Haemophilus influenzae*, *Escherichia coli*, *Candida albicans*, and *Aspergillus niger*.

The main compounds determined in apple by-products with ranges of their concentrations are listed in Table 14.

Many have been written about the application of apple pomace itself. However, the present work concerns the properties and application of bioactive compounds derived from apple pomace. The newest studies reported valuable activities and interesting applications of phytochemicals from apple pomace are listed in Table 15. Preclinical studies have found apple pomace extracts and isolated compounds improved lipid metabolism, antioxidant status, and gastrointestinal function and had a positive effect on metabolic disorders (e.g., hyperglycemia, insulin resistance, etc.) [193]. As was reported by Gołębiewska et al. [194], despite medicine and cosmetics, apple pomace phytochemicals found recent applications in building and construction industries as green corrosion inhibitors and wood protectors [194].

Phenolic content is related to the antioxidant properties of apple pomace, and procyanidins are considered the major contributors to the antioxidant capacity of apples. Despite high concentrations in apples, catechins and procyanidins are very often absent in the extract from apple pomace. The exposure of polyphenols to polyphenoloxidase during apple processing caused, in addition to native apple phytochemicals, their oxidation products also represent a significant part of the overall polyphenolic fraction. Moreover, the polyphenols can interact non-covalently with polysaccharides; thus, they become non-extractable. Fernandes et al. [178] reported that such complexes represented up to 40% of the available polyphenols from apple pomace, potentially relevant for agro-food waste valuation. Moreover, it has been revealed that the use of appropriate extraction procedures, such as microwave-superheated water extraction (MWE) of the hot water/acetone, as well as additional hydrolysis, made it possible to recover these valuable compounds from apple pomace. This knowledge will allow for designing more diversified solutions for agro-food waste valuation [178]. The strong antioxidant in apple pomace is quercetin, which has protective effects against breast and colon cancer, as well as heart and liver diseases [203].

Apple is a unique plant in the *Rosaceae* family due to the high content of phloridzin, a major phenolic compound in commercial varieties of apples [203]. Phloridzin has anti-diabetic potential and could be applied as a natural sweetening agent [200]. Phloridzin from apple waste was also tested as the substrate for the production of food dye through its enzymatic oxidation. The yellow product, so-called phloridzin oxidation products (POP), turned out to be a good alternative to tartrazine and other potentially toxic food yellow pigments [200,201].

Interesting phytochemicals of apple pomace are triterpenoids, particularly ursolic acid. It has attracted attention because of its therapeutic potential associated with several functional properties such as antibacterial, antiprotozoal, anti-inflammatory, and antitumor [196]. Woźniak et al. [190] optimized the method of its extraction using supercritical carbon dioxide. The data obtained allowed the prediction of the extraction curve for the process conducted on a larger scale.

As has been mentioned previously, apple pomace contains some amount of seeds. Walia et al. [192] proved that also apple seed oil could be a promising raw material for the production of natural antioxidants and anticancer agents. The authors tested the fatty acid composition and physicochemical and antioxidant properties of oil extracted from apple seeds separated from industrial pomace. The dominant fatty acids were oleic acid (46.50%) and linoleic acid (43.81%).

The major constituent in apple seed is also amygdalin, which may be metabolized to toxic hydrogen cyanide [203,204]. However, in the literature, there are also several reports of the positive pharmacological activity of amygdalin. Luo et al. [205] showed its anti-fibrotic properties in the case of liver fibrosis. Song and Xu [206] proved that amygdalin exhibits analgesic effects in mice, probably by inhibiting prostaglandins E2 and nitric oxide synthesis. Despite so many above reports, there is still a need for human and animal studies to confirm the protection against the disease’s effects of apple pomace.

### 2.8. Winery Waste

The major winery by-products are grape pomace and marc, including seeds, pulp, skins, stems, and leaves. Bioactive phytochemicals present in residues from wine-making are mainly represented by polyphenols belonging to various groups of compounds, such as phenolic acids (hydroxybenzoic acids and hydroxycinnamic acids), flavonoids (flavanols or flavan-3-ols, anthocyanins, proanthocyanidins, flavones, and flavonols), and stilbenes and anthocyanins. The relative concentrations of the different phenolic compounds are influenced by genotype (red or white grapes), a distinct fraction of residues, as well as agro-climatic conditions [207]. The presence of polyphenolic compounds in grape residues supports the potential of the investigation and valorization of this agro-industrial waste. The compounds identified in grapes by-products with their concentrations are listed in Table 16.

The residues derived from the grape processing contain phytochemicals of interest for the production of preservatives, dyes, enriched foods, medicines, and products aimed at personal care, pharmaceutical, and cosmetic industries. The presence of bioactive compounds with antioxidant, antimicrobial, anti-inflammatory, anti-tumor, and protective activity of the cardiovascular system provides possibilities for many applications [221]. The potential beneficial role of phytochemicals of grape pomace in the prevention of disorders associated with oxidative stress and inflammation, such as endothelial dysfunction, hypertension, hyperglycemia, diabetes, and obesity, is due to the mechanisms concerned especially modulation of antioxidant/prooxidant activity, improvement of nitric oxide bioavailability, reduction of pro-inflammatory cytokines and modulation of antioxidant/inflammatory signal pathways [222].

It has been proven that the antioxidant properties of polyphenols in grape pomace help to prevent radical oxidation of the polyunsaturated fatty acids of low-density lipoproteins (LDL) and hence, are conducive to the prevention of cardiovascular diseases [223]. The compounds derived from grape pomace were also tested for their anti-inflammatory and anti-carcinogenic effect [224]. Álvarez et al. [225] studied the impact of procyanidins from grape pomace as inhibitors of human endothelial NADPH oxidase and stated the decrease in the production of reactive oxygen species. A rich source of procyanidins is grape seeds. They are widely consumed in some countries in the form of powder as a dietary supplement because of several related health benefits associated with procyanidins. They present antitumor-promoting activity, inhibit growth and induce apoptosis in human prostate cancer cells, as well as significantly reducing atherosclerosis in the aorta.

Seeds contain a very broad spectrum of procyanidins, with the dominant compounds being the dimers, trimers, and tetramers of catechin or epicatechin. Higher polymers are also present but at much lower abundance. Besides, every polymer can also be found as a gallic acid ester.

Very important is the anti-microbial activity of bioactive compounds included in grapes wastes. Mendoza et al. [226] demonstrated the antifungal properties of extracts from winery by-products against *Botrytis cinerea*, the causal agent of gray mold, considered the most important pathogen responsible for postharvest decay of fresh fruit and vegetables. Moreover, a few reports are available in the literature about the effective action of polyphenol-rich extracts from vinification by-products against various pathogenic bacteria and insects, e.g., *Listeria monocytogenes*, *Leptinotarsa decemlineata*, and *Spodoptera littoralis* [1]. The potential health benefits of plant phenolics cause much interest and consideration in a lot of agri-food applications for phenolics extracted from grape wastes [16]. There are a lot of studies on the application of phytochemicals from grape pomace in the meat industry [221].

To facilitate the industrial application of wine waste polyphenols, encapsulation was recently developed to improve the stability of valuable compounds in different conditions of light and temperature [227,228]. 

The examples of the newest potential applications and valuable properties of phytochemicals derived from winery waste are listed in Table 17.

### 2.9. Citrus Residues

Citrus fruits from the family *Rutaceae* include oranges, lemons, limes, grapefruits, mandarins, and tangerines. They are well known for their nutritional value, as they are good sources of dietary fiber, pectin, vitamin C, vitamin B group, carotenoids, flavonoids, and limonoids (Table 18). It is estimated that approximately 140 chemical components have been isolated and identified from citrus peels, and flavonoids are the main group of phytochemicals with biological activity [245]. Afsharnezhad et al. [165] evaluated the antioxidant potential of extract from various fruit peels and stated that the maximum DPPH radical scavenging activity, total phenols, and total anthocyanins were observed in orange peels.

Citrus peels are widely used by-products for the production of essential oils, which have great commercial importance due to their aroma, antifungal and antimicrobial properties. Citrus essential oil is employed in the food industry, perfumes, cosmetics, domestic household products, and pharmaceuticals [257]. The main ingredient is limonene, accounting for more than 94% of citrus essential oil [258]. It is used as an insect-killing agent in pesticides and a good biodegradable and non-toxic solvent [257]. Furthermore, limonene has shown regulatory effects on neurotransmitters and stimulant-induced changes in dopamine neurotransmission [258].

The citrus waste contained high amounts of organic and phenolic acids, as well as flavonoids. Among flavonoids, the main compounds are flavanones and flavones (such as naringenin, hesperetin, and apigenin glycosides) as well as polymethoxylated flavones (PMFs), not found in other fruit species [259,260]. Okino Delgado and Feuri [258] indicated that polymethoxylated flavones, at a dosage of 250 mg/kg, exhibit an anti-inflammatory effect comparable to ibuprofen. The most widely studied PMFs are tangeretin and nobiletin. They are exclusively derived from citrus peels. Lv et al. [261] stated that nobiletin and its derivatives showed anti-cancer activity. Generally, anticancer activity increases with the increasing number of methoxy groups because PMFs have then higher hydrophobicity for approaching and penetrating cancer cells [244]. Moreover, PMFs exhibit a broad spectrum of other biological activities such as anti-obesity, anti-atherosclerosis, antiviral and antioxidant properties [262,263].

Among flavanones, citrus peel is rich in eriocitrin, hesperidin, diosmin, neohesperidin, didymin, and naringin. Chiechio et al. [264] used red orange and lemon extract rich in flavanones for in vivo assays on male CD1 mice fed with a high-fat diet. The results showed that an 8-week treatment with the extract was able to induce a significant reduction in glucose, cholesterol, and triglyceride levels in the blood, with positive effects on the regulation of hyperglycemia and lipid metabolism. Barbosa et al. [265] tested flavanones obtained from citrus pomace by enzyme-assisted and conventional hydroalcoholic extraction as an agent against *Salmonella enterica* subsp. *enterica*. Tested extracts decreased the expression of genes associated with cell invasion. Moreover, the results suggest that extracts and flavanones inhibit *Salmonella Typhimurium* adhesion by interacting with fimbriae and flagella structures and downregulating fimbrial and virulence genes.

Citrus peels also contained some flavonols, such as rutin, isorhamnetin 3-*O*-rutinoside, quercetin-*O*-glucoside, and myricetin, as well as phenolic acids, but at a much lower concentration. It has been proven that *Citrus reticulata* waste extract, mainly including rutin, was the most effective against gram-negative bacteria and the three pathogenesis fungi: *Bacillus subtilis*, *Candida albicans* and *Aspergillus flavus* [266].

Citrus seeds are also a good source of valuable components, particularly oil rich in carotenoids (19.01 mg/kg), phenolic compounds (4.43 g/kg), tocopherols (135.65 mg/kg) and phytosterols (1304.2 mg/kg) [251]. This oil was characterized by high antioxidant activity ranging from 56.0% to 70.2%. 

A summary of the main phytochemical constituents, together with their concentrations in citrus residues, as well as their newest applications and properties, is presented in Table 18 and Table 19, respectively.

### 2.10. Olive Waste

The cultivation of olive trees is a widespread practice in the Mediterranean region, accounting for about 98% of the world’s olive cultivation. A large number of phenolic compounds occur in both olive oil and olive waste that includes both leaves and the residues of oil production [275,276]. Their chemical characterization was reported by Dermeche et al. [277]. The main groups of phenolic compounds in olive mill wastes are phenolic acids, secoiridoids, and flavonoids, and the most abundant polyphenols are oleuropein, hydroxytyrosol, verbascoside, apigenin-7-glucoside, and luteolin-7-glucoside [278] (Table 20). Olive mill wastewater obtained during oil production is a complex mixture of vegetation waters and processing waste of the olive fruit; it is characterized by a dark color, strong odor, a mildly acidic pH, and a very high inorganic and organic load [279]. The organic fraction consists essentially of sugars, tannins, polyphenols, polyalcohols, proteins, organic acids, pectins and lipids [277]. About 30 million m^3^ of olive mill wastewater are produced annually in the world as a by-product of the olive oil extraction process; because of the high polyphenolic content (0.5–24 g/L), this by-product is difficult to biodegrade and a relevant environmental and economic issue [280].

Polyphenols also occur in the leaves [287]. These compounds confer bioactive properties on olive leaf extracts, such as antioxidant, antimicrobial, and antitumor activity; the capacity to reduce the risk of coronary heart disease was also reported [288]. Olive leaves can be collected as a by-product during oil processing (about 10% of the total weight of the olives) but can also be a residue of olive tree pruning. Some authors estimated that about 25 kg of by-products (twigs and leaves) could be obtained annually by pruning per tree [289]. To date, this by-product is often used as animal feed, even if this natural resource rich in antioxidant phenolic compounds should be valorized [290].

The qualitative and quantitative content of phenolic compounds is often heterogeneous in olive by-products; however, several studies reported the bioactive properties of these phenolic compounds, promising potential as antioxidant, anti-inflammatory, and antimicrobial agents. The antioxidant activities of olive mill wastewater and olive pomace have been demonstrated by different antioxidant assays as DPPH radical-scavenging activity, superoxide anion scavenging, LDL oxidation, and the protection of catalase against hypochlorous acid [281,291,292]. An overview of the pharmacology of olive oil and its active ingredients has been reported by Visioli et al. [293]. Recently, a novel stable ophthalmic hydrogel containing a polyphenolic fraction obtained from olive mill wastewater was formulated [294]. Among olive polyphenols, hydroxytyrosol is one of the main phenolic compounds; it can occur in its free form or as secoiridoids (oleuropein and its aglycone). For its polarity, it is more abundant in olive mill wastewater and pomace rather than in olive oil. Anticancer, antioxidant, and anti-inflammatory properties have been reported for hydroxytyrosol [295,296]. In vitro antioxidant and skin regenerative properties have been reported by Benincasa et al. [297].

Moreover, the polyphenol fraction obtained from olive mill wastewater showed activities against bacteria, fungi, plants, animals, and human cells; antibacterial activities against several bacterial species (*Staphylococcus aureus*, *Bacillus subtilis*, *Escherichia coli* and *Pseudomonas aeruginosa*) have been reported by Obied et al. [298]. Fungicidal activities have also been reported [299]. Moreover, the effects of phenolic compounds from olive waste on *Aspergillus flavus* growth and aflatoxin B_1_ production were investigated [300,301]. The olive mill wastewater polyphenols did not inhibit the *Aspergillus flavus* fungal growth rate but significantly reduced the aflatoxin B_1_ production (ranging from 88 to 100%) at 15% concentration [302].

Finally, cytoprotection of brain cells by olive mill wastewater has been studied by Schaffer et al. [303]. The cytoprotective effects were correlated to the content of hydroxytyrosol. 

These studies showed the numerous beneficial and bioactive activities of polyphenols fraction obtained by olive by-products; for their use, it is often carried out an appropriate fractionation and/or purification to control their concentration and to avoid some antagonist effects.

Various valuable properties and the newest studies on the application of biologically active compounds derived form olive waste are presented in Table 21.

## 3. Conclusions

The ever-increasing amount of processed food raw materials entails an increasing amount of biowaste. Their management has become a growing problem. The consulted literature shows that discussed waste still contains valuable ingredients, medicinally important phytochemicals, and good antioxidants, so it is very important to valorize them. Currently, the recovery of different valuable phytochemicals from agro-industrial waste has become an imperative research area among the scientific community because agro-industrial residues of plant materials are a cheap and natural source of bioactive compounds, which can be used in the prevention and treatment of various diseases. Despite many studies on the valuable properties and potential applications, still, not many solutions are implemented in the industry. This is probably caused by legislation that can affect the valorization of such waste biomass. There are not many regulatory and legal provisions for their use. In the European Union, the use of agricultural residues as food ingredients is regulated by the European Community Regulation (EC) No 178/2002. However, in order to use them as natural additives, proper authorization as a novel food is necessary (Regulation (EC) No 2015/2283) [304]. There is no doubt that the industrial application of the extracts needs to be regulated.

According to the circular bioeconomy and biorefinery concept, food waste should be recycled inside the whole food value chain from field to fork in order to formulate functional foods and nutraceuticals. Nonetheless, it is important to implement environmentally friendly industrial extraction procedures. Moreover, despite so many above reports, there is still a need for human and animal studies, as well as studies in the field in the case of plants, to confirm the protective effect of such phytochemicals against diseases. 

Taking into account the European Union’s emphasis on the development of a circular economy and reducing the carbon footprint, it is expected that the effective application of these wastes will be carried out and that regulations will be developed in accordance with needs.

## Figures and Tables

**Table 1:** Amount of residues from some crops produced in the world in 2020.

Crop	Global Crop Production * [Million Ton]	Residue to Crop Ratio	Amount of Residue ** [Million Ton]	References
Sugarcane	1869.7	0.1	189.1	Jiang et al. [9]
Maize	1162.4	2.0	2324.8	Jiang et al. [9]
Wheat	760.9	1.18	897.9	Searle and Malins [10]
Rice	756.7	1.0	756.7	Jiang et al. [9]
Potato	359.1	0.4	143.6	Ben Taher et al. [11]
Soybean	353.5	1.5	530.3	Yanli et al. [12]
Sugar beet	253.0	0.27	68.3	Searle and Malins [10]
Tomato	186.8	3.5	653.8	Oleszek et al. [13]
Barley	157.0	1.18	185.3	Searle and Malins [10]
Banana	119.8	0.6	71.9	Gabhane et al. [14]
Cucumber	91.3	4.5	410.9	Oleszek et al. [13]
Apples	86.4	0.25	21.6	Cruz et al. [15]
Grapes	78.0	0.3	23.4	Muhlack et al. [16]
Oranges	75.5	0.5	37.8	Rezzadori et al. [17]
Olives	23.6	0.12	2.8	Searle and Malins [10]

* based on FAOSTAT, 2022, ** calculated based on the global crop production in 2020 and the residue-to-crop ratio according to cited references.

**Table 2:** Phytochemicals derived from sugarcane bagasse.

Name	MW * [g mol^−1^]	C_x_H_y_O_z_	References
Phenolic acids—hydroxybenzoic acids			
*p*-Hydroxybenzoic acid	138.12	C_7_H_6_O_3_	Zheng et al. [19]
Vanillic acid	168.14	C_8_H_8_O_4_	Zheng et al. [19]
Benzoic acid	122.12	C_7_H_6_O_2_	Zheng et al. [19]
Protocatechuic acid	154.12	C_7_H_6_O_4_	Zheng et al. [19]
Gallic acid	170.12	C_7_H_6_O_5_	Zhao et al. [26]
Syringic acid	198.17	C_9_H_10_O_5_	Zhao et al. [26]
Phenolic acids—hydroxycinnamic acids			
*p*-Coumaric acid	164.04	C_9_H_8_O_3_	González–Bautista et al. [28]
Cinnamic acid	148.16	C_9_H_8_O_2_	González–Bautista et al. [28]
Ferulic acid	194.18	C_10_H_10_O_4_	González–Bautista et al. [28]
Caffeic acid	180.16	C_9_H_8_O_4_	González–Bautista et al. [28]
Chlorogenic acids	354.31	C_16_H_18_O_9_	Zhao et al. [26]
Sinapic acid	224.21	C_11_H_12_O_5_	Zhao et al. [26]
Flavonoids—flavonols			
Quercetin	302.24	C_15_H_10_O_7_	Zheng et al. [19]
Flavonoids—flavones			
Luteolin	286.24	C_15_H_10_O_6_	Zheng et al. [29]
Tricin	330.29	C_17_H_14_O_7_	Zheng et al. [29]
Flavonoid glycosides			
Diosmetin 6-*C*-glucoside	462.40	C_22_H_22_O_11_	Zheng et al. [29]
Tricin 7-*O*-*β*-glucopyranoside	492.43	C_23_H_24_O_12_	Zheng et al. [29]
Isoflavone			
Genistin	432.37	C_21_H_20_O_10_	Zheng et al. [19]
Genistein	270.24	C_15_H_10_O_5_	Zheng et al. [19]
Others			
Catechol	110.11	C_6_H_6_O_2_	Zheng et al. [19]
Phenol	94.11	C_6_H_6_O	Zheng et al. [19]
Guaiacol	124.14	C_7_H_8_O_2_	Zheng et al. [19]
Vanillin	152.15	C_8_H_8_O_3_	Zheng et al. [19]
Isovanillin	152.15	C_8_H_8_O_3_	Van der Pol et al. [30]
Syringaldehyde	182.17	C_9_H_10_O_4_	Zheng et al. [19]
Piceol	136.15	C_8_H_8_O_2_	Van der Pol et al. [30]
Apocynin	166.17	C_9_H_10_O_3_	Van der Pol et al. [30]
Acetosyringone	196.19	C_10_H_12_O_4_	Van der Pol et al. [30]
Syringaldehyde	182.17	C_9_H_10_O_4_	Van der Pol et al. [30]
Creosol	138.16	C_8_H_10_O_2_	Lv et al. [31]
4-Ethylguaiacol	152.19	C_9_H_12_O_2_	Lv et al. [31]
Chavicol	134.17	C_9_H_10_O	Lv et al. [31]
4-Vinylguaiacol	150.17	C_9_H_10_O_2_	Lv et al. [31]
4-Allylsyringol	194.23	C_11_H_14_O_3_	Lv et al. [31]

* MW—molecular weight.

**Table 3:** Biological activities and potential applications of phytochemicals obtained from sugarcane bagasse.

Material	Extract/Compound	Biological Activity/Application	References
Sugarcane bagasse	phenolic compounds	- natural antioxidant - used in pharmacology	Al Arni et al. [27]
		- antibacterial agents against the foodborne pathogens *Escherichia coli*, *Listeria monocytogenes*, *Staphylococcus aureus*, *Salmonella typhimurium*	Zhao et al. [26]
	gallic and tannic acids	- deactivate cellulolytic and hemicellulolytic enzymes	Michelin et al. [32]
	extract	- antioxidant and radical scavenging activity - antimicrobial activity against *Sta*- *phylococcus aureus* TISTR029 and *Escherichia coli* O157:H7 - added value for the sugar industry	Juttuporn et al. [33]
		- antihyperglycemic ability - useful therapeutic agents to treat T2D patients	Zheng et al. [19]
		- used for the low-cost bio-oil production	Treedet and Suntivarakorn [34]
		- feedstock for ethanol (bioethanol) production	Krishnan et al. [35] Zhu et al. [36]
		- raw material for the production of industrial enzymes, xylose, glucose, methane	Guilherme et al. [37]
		- raw material for the production of xylitol and organic acids	Chandel et al. [38]
		- used to prepare highly valued succinic acid	Xi et al. [23]
		- used as a reducing agent in synthesizing biogenic platinum nanoparticles	Ishak et al. [20]
		- used as a fuel to power sugar mills	Mohan et al. [22]

**Table 4:** Phytochemicals identified in corn waste.

Name	MW [g mol^−1^]	Molecular Formula	References
Phenolic acids—hydroxycinnamic acids			
*p*-Coumaric acid	164.04	C_9_H_8_O_3_	Guo et al. [39]
Ferulic acid	194.18	C_10_H_10_O_4_	Guo et al. [39]
trans-ferulic acid	194.18	C_10_H_10_O_4_	Guo et al. [39]
trans-ferulic acid methyl ester	208.21	C_11_H_12_O_4_	Guo et al. [39]
cis-ferulic acid	194.18	C_10_H_10_O_4_	Guo et al. [39]
cis-ferulic acid methyl ester	208.21	C_11_H_12_O_4_	Guo et al. [39]
Flavonoids—flavonols			
Rutin	610.52	C_27_H_30_O_16_	Bujang et al. [40]
Quercetin-3-*O*-glucoside	463.37	C_21_H_19_O_12_	Dong et al. [41]
Isorhamnetin-3-*O*-glucoside	478.41	C_22_H_22_O_12_	Dong et al. [41]
Kaempferol-3-*O*-glucoside	447.37	C_21_H_19_O_11_	Li et al. [42]
Maysin	576.50	C_27_H_28_O_14_	Haslina and Eva [43]
Isoorientin-2″-*O*-*α*-l-rhamnoside	594.50	C_27_H_30_O_15_	Haslina and Eva [43]
Maysin-3′-methyl ether	590.50	C_28_H_30_O_15_	Tian et al. [44]
ax-4″–OH–3′-Methoxymaysin	592.50	C_28_H_32_O_14_	Tian et al. [44]
2″-*O*-*α*-l-Rhamnosyl-6-C-fucosylluteolin	578.50	C_27_H_30_O_14_	Tian et al. [44]
Flavonoids—anthocyanins			
Pelargonidin-3-*O*-glucoside	433.40	C_21_H_21_O_10_	Lao and Giusti [45]
Pelargonidin-3-(6″malonylglucoside)	519.23	C_24_H_23_O_13_	Chen et al. [46]
Cyanidin-3-*O*-glucoside	449.39	C_21_H_21_O_11_	Barba et al. [47]
Cyanidin 3-(6″-malonylglucoside)	535.11	C_24_H_23_O_14_	Fernandez-Aulis et al. [48]
Peonidin-3-*O*-glucoside	463.41	C_22_H_23_O_11_	Barba et al. [47]
Peonidin-3-(6″malonylglucoside)	549.50	C_25_H_25_O_14_	Fernandez-Aulis et al. [48]
Other compounds			
*p*-Hydroxybenzaldehyde	122.12	C_7_H_6_O_2_	Guo et al. [39]
*β*-Sitosterol glucoside	576.85	C_35_H_60_O_6_	Guo et al. [39]
Indole-3-acetic acid	175.06	C_10_H_9_NO_2_	Wille and Berhow [49]
Vanillin	154.05	C_8_H_8_O_3_	Guo et al. [39]

**Table 5:** Biological activity and potential applications of phytochemicals obtained from corn wastes.

Material	Extract/Compound	Biological Activity/Application	References
Corn bran	tocopherols and polyphenolic compounds	- antioxidant properties - used as bioactive compounds in cosmetics or natural substitutes (antioxidants, preservatives, stabilizers, emulsifiers, and colorings) in foods to prevent potential adverse effects associated with the consumption of artificial ingredients	Galanakis [62]
Corn husk	extract	- used in the treatment of diabetes because it has shown high: - antidiabetic potential	Brobbey et al. [51]
		- anti-inflammatory effects	Roh et al. [63]
Corn stigma	extract	- antifungal and antibacterial activities against 23 of the studied microorganisms - use as a functional ingredient in the food and pharmaceutical industry	Boeira et al. [64]
Corn tassel	extract	- used as a traditional medicine in China - antioxidant capacity - the high ability to inhibit the proliferation of MGC80-3 gastric cancer cells	Wang et al. [65]
	tasselin A	- inhibition of melanin production - used as an ingredient in skin care whitener	Wille and Berhow [49]
Corn pollen	phenolic compounds	- antiradical activity	Bujang et al. [40]
	extract	- the source of functional and bioactive compounds for the nutraceutical and pharmaceutical industries	Bujang et al. [40]
		- the source of antioxidants and is high in nutrients	Žilić et al. [58]

**Table 6:** Phytochemicals identified in potato waste.

Name	MW [g mol^−1^]	Molecular Formula	References
Phenolic acids—hydroxycinnamic acids			
*p*-Coumaric acid	164.04	C_9_H_8_O_3_	Frontuto et al. [71]
Ferulic acid	194.18	C_10_H_10_O_4_	Javed et al. [72]
Caffeic acid	180.16	C_9_H_8_O_4_	Samarin et al. [73]
Chlorogenic acid	354.31	C_16_H_18_O_9_	Javed et al. [72]
Sinapic acid	224.21	C_11_H_12_O_5_	Mohdaly et al. [67]
Cinnamic acid	148.16	C_9_H_8_O_2_	Mohdaly et al. [67]
Phenolic acids—hydroxybenzoic acids			
Gallic acid	170.12	C_7_H_6_O_5_	Javed et al. [72]
Vanillic acid	168.15	C_8_H_8_O_4_	Javed et al. [72]
Protocatechic acid	154.12	C_7_H_6_O_4_	Frontuto et al. [71]
*p*-Hydroxybenzoic acid	138.12	C_7_H_6_O_3_	Chamorro et al. [74]
3-Hydroxybenzoic acid	138.12	C_7_H_6_O_3_	Paniagua–García et al. [75]
4-Hydroxybenzoic acid	138.12	C_7_H_6_O_3_	Paniagua–García et al. [75]
2,5-Dihydroxybenzoic acid	154.12	C_7_H_6_O_4_	Paniagua–García et al. [75]
Syringic acid	198.17	C_9_H_10_O_5_	Sarwari et al. [76]
Cyclohexanecarboxylic acids			
Quinic acid	192.17	C_7_H_12_O_6_	Wu et al. [77]
Flavonoids—flavonols			
Rutin	610.52	C_27_H_30_O_16_	Silva–Beltran et al. [78]
Quercetin	302.24	C_15_H_10_O_7_	Silva–Beltran et al. [78]
Flavonoids—anthocyanin			
Pelargonidin-3-(*p*-coumaryoly rutinoside)- 5-glucoside	919.81	C_42_H_47_O_23_	Chen et al. [79]
Petunidin-3-(*p*-coumaroyl rutinoside)- 5-glucoside	933.86	C_43_H_49_O_23_	Chen et al. [79]
Alkaloids			
*α*-Chaconine	852.06	C_45_H_73_NO_14_	Ji et al. [80]
*α*-Solanine	868.06	C_45_H_73_NO_15_	Ji et al. [80]
Solanidine	397.64	C_27_H_43_NO	Hossain et al. [81]
Demissidine	399.65	C_27_H_45_NO	Hossain et al. [81]
Commersonine	1048.20	C_51_H_85_NO_21_	Rodríguez–Martínez et al. [82]
*α*-Tomatine	1034.19	C_50_H_83_NO_21_	Rodríguez–Martínez et al. [82]

**Table 7:** Biological activity and potential applications of phytochemicals obtained from potato wastes.

Material	Extract/Compound	Biological Activity/Application	References
Potato peel	phenolic compounds	- antioxidant activity	Singh et al. [91] Albishi et al. [83]
		- used as a food preservative - pharmaceutical ingredient	Maldonado et al. [92]
	extract	- natural food additives as an antioxidant for fresh-cut fruits	Akyol et al. [93] Venturi et al. [94]
		- food preservative - pharmaceutical ingredient	Gebrechristos and Chen [95]
		- limit oil oxidation	Amado et al. [96]
		- hepatoprotective effects, - protects erythrocytes against oxidative damage - lowers the toxicity of cholesterol oxidation products - attenuate diabetic alterations	Hsieh et al. [97]
		- protects atopic dermatitis	Yang et al. [98]
		- amylase and feed-stock for bioethanol production	Khawla et al. [99]
		- antioxidant, antibacterial, apoptotic, chemopreventive and anti-inflammatory	Wu [100]
		- bio-oil production	Liang et al. [101]
		- production of bacterial cellulose - biopolymer production	Abdelraof et al. [102]
		- antiobesity properties - used in the production of antiobesity functional food	Elkahoui et al. [103] Chimonyo [104]
		- a source of natural antioxidants against human enteric viruses (antiviral effect on the inhibition of Av-05 and MS2 bacteriophages, which were used as human enteric viral surrogates)	Silva-Beltran et al. [78]
	freeze-dried aqueous extracts	- use as food additives	Singh et al. [91]
	glycoalkaloids	- the potential of being used by the pharmaceutical industry	Apel et al. [105]
Potato waste	extract	- as additives to biscuit	Khan et al. [106]
	glycoalkaloids	- precursors for the production of hormones, antibiotics and anticancer drugs - precursors for neurological and gastrointestinal disorders - anti-cancer and anti-proliferative activities in vitro	Hossain et al. [81] Hossain et al. [87] Ding et al. [107] Alves–Filho et al. [86]
	steroidal alkaloids	- biological properties such as antimicrobial, anti-inflammatory and anticarcinogenic activities	Kenny et al. [108]

**Table 8:** Phytochemicals identified and quantified in soybean waste.

Name	Soybean Residue	MW [g mol^−1^]	C_x_H_y_O_z_	Concentration	References
Phenolic acids—hydroxybenzoic acids					
*p*-Hydroxybenzoic acid	herb root meal	138.12	C_7_H_6_O_3_	22.2–38.3 ^a,b^ 4.1–32.5 ^a,b^ 51 ^a^	Šibul et al. [113] Šibul et al. [113] Freitas et al. [120]
Salicylic acid	meal	138.12	C_7_H_6_O_3_	38 ^a^	Freitas et al. [120]
Protocatechuic acid	herb root	154.12	C_7_H_6_O_4_	4.4–14.4 ^a,b^ 2.35–4.71 ^a,b^	Šibul et al. [113]
Gentisic acid	herb root	154.12	C_7_H_6_O_4_	<0.08–4.78 ^a,b^ <0.08–7.17 ^a,b^	Šibul et al. [113]
Vanillic acid	herb root meal	168.14	C_8_H_8_O_4_	<0.4–44.9 ^a,b^ 43.0–75.2 ^a,b^ 91 ^a^	Šibul et al. [113] Freitas et al. [120]
Syringic acid	herb root meal	198.17	C_9_H_10_O_5_	12.0–14.2 ^a,b^ 20.6–42.0 ^a,b^ 81 ^a^	Šibul et al. [113] Freitas et al. [120]
Gallic acid	meal	170.12	C_7_H_6_O_5_	77 ^a^	Freitas et al. [120]
Phenolic acids—hydroxycinnamic acids					
*p*-Coumaric acid	herb root meal	164.04	C_9_H_8_O_3_	7.45–14.5 ^a,b^ 1.61–2.89 ^a,b^ 20 ^a^	Šibul et al. [113] Freitas et al. [120]
Ferulic acid	herb root meal	194.18	C_10_H_10_O_4_	5.89–14.0 ^a,b^ 4.55–7.66 ^a,b^ 3 ^a^	Šibul et al. [113] Freitas et al. [120]
Caffeic acid	herb root meal	180.16	C_9_H_8_O_4_	14.2–24.9 ^a,b^ <0.08 ^a^ 61 ^a^	Šibul et al. [113] Freitas et al. [120]
Sinapic acid	meal	224.21	C_11_H_12_O_5_	27 ^a^	Freitas et al. [120]
Cyclohexanecarboxylic acids					
Quinic acid	herb root	192.17	C_7_H_12_O_6_	399–532 ^a,b^ 111–249 ^a,b^	Šibul et al. [113]
5-*O*-Caffeoylquinic acid	herb root meal	354.31	C_16_H_18_O_9_	<8–235 ^a,b^ <8 ^a^ 35 ^a^	Šibul et al. [113] Freitas et al. [120]
Flavonoids—flavonols					
Kaempferol	herb root meal	286.23	C_15_H_10_O_6_	<16–21.1 ^a,b^ <16 ^a^ 4 ^a^	Šibul et al. [113] Freitas et al. [120]
Quercetin	herb root	302.24	C_15_H_10_O_7_	<16–278 ^a,b^ <16 ^a^	Šibul et al. [113]
Isorhamnetin	herb root	316.26	C_16_H_12_O_7_	<40–159 ^a,b^ <40 ^a^	Šibul et al. [113]
Quercitrin	herb root	448.38	C_21_H_20_O_11_	<0.06 ^a^ <0.06 ^a^	Šibul et al. [113]
Kaempferol 3-*O*-glucoside	herb root	448.38	C_21_H_20_O_11_	59.3–140 ^a,b^ 1.50–2.64 ^a,b^	Šibul et al. [113]
Hyperoside	herb root	464.38	C_21_H_20_O_12_	<0.1–825 ^a,b^ <0.06 ^a^	Šibul et al. [113]
Quercetin 3-*O*-glucoside	herb root	464.10	C_21_H_20_O_12_	<0.06–967 ^a,b^ <0.06 ^a,b^	Šibul et al. [113]
Rutin	herb root meal	610.52	C_27_H_30_O_16_	7.05–4636 ^a,b^ <2 ^a^ 49 ^a^	Šibul et al. [113] Freitas et al. [120]
Flavonoids—flavones					
Apigenin	herb root	270.24	C_15_H_10_O_5_	17.4–759 ^a,b^ <8–22.3 ^a,b^	Šibul et al. [113]
Baicalein	herb root	270.24	C_15_H_10_O_5_	27.8–745 ^a,b^ <16–24.7 ^a,b^	Šibul et al. [113]
Luteolin	herb root	286.24	C_15_H_10_O_6_	<40–194 ^a,b^ <40 ^a^	Šibul et al. [113]
Chrysoeriol	herb root	300.26	C_16_H_12_O_6_	<4–9.57 ^a,b^ <4 ^a^	Šibul et al. [113]
Vitexin	herb root	432.38	C_21_H_20_O_10_	1.37–2.36 ^a,b^ 1.81–3.57 ^a,b^	Šibul et al. [113]
Apigenin 7-*O*-glucoside	herb root	432.38	C_21_H_20_O_10_	14.3–261 ^a,b^ <0.2–1.99 ^a,b^	Šibul et al. [113]
Luteolin 7-*O*-glucoside	herb root	448.37	C_21_H_20_O_11_	<4–145 ^a,b^ <4 ^a^	Šibul et al. [113]
Apiin	herb root	564.49	C_26_H_28_O_14_	<0.06–20.8 ^a,b^ <0.06 ^a^	Šibul et al. [113]
Flavonoids—flavanones					
Naringenin	herb root meal	272.26	C_15_H_12_O_5_	3.46–8.46 ^a,b^ 6.52–15.9 ^a,b^ 25 ^a^	Šibul et al. [113] Freitas et al. [120]
Hesperidin	meal	610.19	C_28_H_34_O_15_	91 ^a^	Freitas et al. [120]
Flavonoids—flavanols					
Catechin	herb root	290.27	C_15_H_14_O_6_	<0.4 ^a^ <0.4 ^a^	Šibul et al. [113]
Epicatechin	herb root	290.27	C_15_H_14_O_6_	<0.4 ^a^ <0.4–36.3 ^a,b^	Šibul et al. [113]
Isoflavones					
Daidzin	okara meal	416.38	C_21_H_20_O_9_	920–1530 ^b,c^ 350 ^a^	Anjum et al. [109] Freitas et al. [120]
Daidzein	okara herb root meal	254.23	C_15_H_10_O_4_	310–639 ^b,c^ 40.7–122 ^a,b^ 40.5–1702 ^a,b^ 30 ^a^	Anjum et al. [109] Šibul et al. [113] Freitas et al. [120]
Genistin	okara meal	432.37	C_21_H_20_O_10_	3280–8360 ^b,c^ 490 ^a^	Anjum et al. [109] Freitas et al. [120]
Genistein	okara herb root meal	270.24	C_15_H_10_O_5_	380–650 ^b,c^ 15.1–39.2 ^a,b^ 159–270 ^a,b^ 50 ^a^	Anjum et al. [109] Šibul et al. [113] Freitas et al. [120]
Glycitin	okara	446.40	C_22_H_22_O_10_	450 ^c^ 160 ^a^	Anjum et al. [109] Freitas et al. [120]
Glycitein	okara meal	284.26	C_16_H_12_O_5_	58 ^c^ 3 ^a^	Anjum et al. [109] Freitas et al. [120]
Saponins					
Soyasaponin B I	meal	943.12	C_48_H_78_O_18_	2510 ^c^	Silva et al. [121]
Soyasaponin B II + III	meal			780 ^c^	Silva et al. [121]

^a^ expressed in mg per kg of dry extract, ^b^ depending on cultivar, ^c^ expressed in mg per kg of residues.

**Table 9:** Biological activity and potential applications of phytochemicals obtained from soybean residues.

Material	Extract/Compound	Biological Activity/Application	References
okara	methanolic and ethanolic extracts	- antioxidant activity - antibacterial activity against *Bacillus subtilis*, *Bacillus megaterium*, *Escherichia coli*, and *Serratia marcescens*	Anjum et al. [109]
pod	Ethanolic extract and its 3 fractions	- antioxidant activity - anticancer activity against human colorectal carcinoma (HCT116) and prostate adenocarcinoma (PC-3)	Pabich et al. [116]
soybean by-product	saponins	- used to remove pesticides residues in fruits and vegetables	Hsu et al. [119]
defatted soy meal	isoflavones	- anti-cancerous, anti-estrogenic, anti-oxidant, anti-inflammatory, and phytoestrogen activities - preventions of cardiovascular and neurological disorders	Wang et al. [122]
soybean by-products	saponins	- insecticidal properties	
soybean meal	aqueous extract	- antioxidant activity - inhibition of lipid peroxidation - antimicrobial activity against several foodborne pathogens - antitumoral activity towards a human glioblastoma cell line	Freitas et al. [120]
soybean cake	soyasapogenol A and its microbial transformation products	- application as anti-inflammatory food supplements	Zhou et al. [123]

**Table 10:** Phytochemicals identified in tomato wastes.

Name	MW [g mol^−1^]	Molecular Formula	References
Phenolic acids—hydroxycinnamic acids			
Chlorogenic acid	354.31	C_16_H_18_O_9_	Bakic et al. [127]
Isochlorogenic acid	354.31	C_16_H_18_O_9_	Szabo et al. [141]
*p*-Coumaric acid	164.16	C_9_H_8_O_3_	Nour et al. [133]
Ferulic acid	194.18	C_10_H_10_O_4_	Perea–Dominguez et al. [131]
Caffeic acid	180.16	C_9_H_8_O_4_	Aires et al. [136]
3,4,5-tricaffeoylquinic acid	678.60	C_34_H_30_O_15_	Szabo et al. [141]
Cinnamic acid	148.16	C_9_H_8_O_2_	Kalogeropoulos et al. [138]
Phloretic acid	166.18	C_9_H_10_O_3_	Kalogeropoulos et al. [138]
Sinapic acid	224.21	C_11_H_12_O_5_	Kalogeropoulos et al. [138]
Rosmarinic acid	360.31	C_18_H_16_O_8_	Ćetković et al. [135]
Phenolic acids—hydroxybenzoic acids			
Gallic acid	170.12	C_7_H_6_O_5_	Nour et al. [133]
Ellagic acid	302.18	C_14_H_6_O_8_	Nour et al. [133]
Vanillic acid	168.15	C_8_H_8_O_4_	Nour et al. [133]
Syringic acid	198.17	C_9_H_10_O_5_	Nour et al. [133]
Protocatechic acid	154.12	C_7_H_6_O_4_	Elbadrawy and Sello [134]
*p*-Hydroxybenzoic acid	138.12	C_7_H_6_O_3_	Kalogeropoulos et al. [138]
Flavonoids			
Quercetin	302.24	C_15_H_10_O_7_	Elbadrawy and Sello [134]
Quercetin-3-*β*-*O*-glucoside	463.40	C_21_H_19_O_12_	Valdez–Morales et al. [142]
Quercetin-3-*O*-sophorosid	626.50	C_27_H_30_O_17_	Kumar et al. [143]
Apigenin-7-*O*-glucoside	432.40	C_21_H_20_O_10_	Concha-Meyer et al. [144]
Isorhamnetin	316.26	C_16_H_12_O_7_	Kumar et al. [143]
Isorhamnetin-3-*O*-gentiobioside	640.50	C_28_H_32_O_17_	Kumar et al. [143]
Rutin	610.52	C_27_H_30_O_16_	Aires et al. [136]
Kaempferol	286.23	C_15_H_10_O_6_	Perea–Dominguez et al. [131]
Kaempferol-3-*O*-rutinoside	394.52	C_27_H_30_O_15_	Aires et al. [136]
Kaempferol-3-*O*-glucoside	447.37	C_21_H_19_O_11_	Kumar et al. [143]
Myricetin	318.24	C_15_H_10_O_8_	Nour et al. [133]
Naringenin	272.26	C_15_H_12_O_5_	Elbadrawy and Sello [134]
Catechin	290.26	C_15_H_14_O_6_	Perea–Dominguez et al. [131]
Epicatechin	290.27	C_15_H_14_O_6_	Kalogeropoulos et al. [138]
Chrysin	254.24	C_15_H_10_O_4_	Kalogeropoulos et al. [138]
Luteolin	286.24	C_15_H_10_O_6_	Kalogeropoulos et al. [138]
Luteolin-7-*O*-glucoside	448.37	C_21_H_20_O_11_	Concha–Meyer et al. [144]
Isoflavones			
Daidzein	254.23	C_15_H_10_O_4_	Kumar et al. [143]
Genistein	270.24	C_15_H_10_O_5_	Kumar et al. [143]
Stilbenes			
Resveratrol	228.24	C_14_H_12_O_3_	Kalogeropoulos et al. [138]
Carotenoids			
Lycopene	536.89	C_40_H_56_	Fritsch et al. [130]
*β*-Carotene	536.89	C_40_H_56_	Kalogeropoulos et al. [138]
Sterols			
*β*-Sitosterol	414.72	C_29_H_50_O	Kalogeropoulos et al. [138]
∆^5^-Avenasterol	412.70	C_29_H_48_O	Kalogeropoulos et al. [138]
Campesterol	400.69	C_28_H_48_O	Kalogeropoulos et al. [138]
Cholestanol	388.70	C_27_H_48_O	Kalogeropoulos et al. [138]
Cholesterol	386.65	C_27_H_46_O	Kalogeropoulos et al. [138]
24-Oxocholesterol	400.60	C_27_H_44_O_2_	Kalogeropoulos et al. [138]
Stigmasterol	412.69	C_29_H_48_O	Kalogeropoulos et al. [138]
Tocopherols			
Tocopherol			Kalogeropoulos et al. [138]
Terpenes			
Squalene	410.73	C_30_H_50_	Kalogeropoulos et al. [138]
Cycloartenol	426.72	C_30_H_50_O	Kalogeropoulos et al. [138]
*β*-Amyrin	426.73	C_30_H_50_O	Kalogeropoulos et al. [138]
Oleanolic acid	456.71	C_30_H_48_O_3_	Kalogeropoulos et al. [138]
Ursolic acid	456.70	C_30_H_48_O_3_	Kalogeropoulos et al. [138]
Palmitic acid	256.43	C_16_H_32_O_2_	Elbadrawy and Sello [134]
Palmitoleic acid	254.41	C_16_H_30_O_2_	Elbadrawy and Sello [134]
Stearic acid	284.48	C_18_H_36_O_2_	Elbadrawy and Sello [134]
Oleic acid	282.47	C_18_H_34_O_2_	Elbadrawy and Sello [134]
Linolenic acid	278.43	C_18_H_30_O_2_	Elbadrawy and Sello [134]
Linoleic acid	280.45	C_18_H_32_O_2_	Elbadrawy and Sello [134]
Myristic acid	228.37	C_14_H_28_O_2_	Elbadrawy and Sello [134]

**Table 11:** Biological activity and potential applications of phytochemicals obtained from tomato wastes.

Material	Extract/Compound	Biological Activity/Application	References
Tomato seeds	polyphenols oil	- antioxidant activity	Zuorro et al. [154]
		- high nutritional quality	Eller et al. [155]
Tomato by-products	extract	- natural antioxidants for the formulation of functional foods or to serve as additives in food systems to elongate their shelf-life - oxidative stability of dairy products - potential nutraceutical resource - animal feed	Savatović et al. [158] Elbadrawy and Sello [134] Nour et al. [159] Abid et al. [160] Ćetković et al. [135] Trombino et al. [161]
Tomato peel	fiber	- food supplement, improving the different chemical, physical and nutritional properties of foods	Navarro–González et al. [137]
	lycopene	- natural color or bioactive ingredient	Ho et al. [162]
	carotenoids	- natural antioxidants and colorants	Horuz and Belibagli [163]

**Table 12:** Phytochemicals identified in banana wastes and their concentration.

Name	Banana Residues	MW [g mol^−1^]	C_x_H_y_O_z_	Concentration	References
Total phenolics				53,800 ^a^	Kabir et al. [166]
				15,180–31,450 ^a,c^	Chaudhry et al. [167]
				29,200 ^a^	Rebello et al. [168]
Total flavonoids				16,440 ^b^	Kabir et al. [166]
				10,800–22,110 ^b,c^	Chaudhry et al. [167]
Phenolic acids—benzoic acids					
Gallic acid	banana peel	170.12	C_7_H_6_O_5_	77.3 ^f^	Behiry et al. [169]
Ellagic acid	banana peel	302.20	C_14_H_6_O_8_	161.9 ^f^	Behiry et al. [169]
Salicylic acid	banana peel	138.121	C_7_H_6_O_3_	2.7 ^f^	Behiry et al. [169]
Phenolic acids—hydroxycinnamic acids					
Chlorogenic acid	banana pseudostem and rhizome	354.31	C_16_H_18_O_9_		Kandasamy et al. [170]
Ferulic acid	red banana peel yellow banana peel banana peel	194.18	C_10_H_10_O_4_	63.55 ^e^ 34.97 ^e^ 16.8 ^f^	Avram et al. [171] Avram et al. [171] Behiry et al. [169]
Sinapic acid	red banana peel yellow banana peel	224.21	C_11_H_12_O_5_	35.17 ^e^ 19.44 ^e^	Avram et al. [171] Avram et al. [171]
Cinnamic acid	banana peel	148.16	C_9_H_8_O_2_	0.7 ^f^	Behiry et al. [169]
o-coumaric acid	banana peel	164.158	C_9_H_8_O_3_	11.2 ^f^	Behiry et al. [169]
Flavonoids—flavonols					
Kaempferol	red banana peel yellow banana peel	286.239	C_15_H_10_O_6_	28.80 ^e^ 9.30 ^e^	Avram et al. [171] Avram et al. [171]
Quercetin	red banana peel yellow banana peel	302.236	C_15_H_10_O_7_	6.14 ^e^ 1.14 ^e^	Avram et al. [171] Avram et al. [171]
Isoqercitrin	red banana peel yellow banana peel	464.096	C_21_H_20_O_12_	10.47 ^e^ 14.54 ^e^	Avram et al. [171] Avram et al. [171]
Rutin	banana peel	610.517	C_27_H_30_O_16_	9730.8 ^f^	Behiry et al. [169]
Myricetin	banana peel	318.235	C_15_H_10_O_8_	115.2 ^f^	Behiry et al. [169]
Myricetin-3-rutinoside	banana peel	626.51	C_27_H_30_O_17_	22.50 ^d^	Behiry et al. [169]
Quercetin-3-rutinoside-3-rhamnoside	banana peel	756.7	C_33_H_40_O_20_	12.91 ^d^	Rebello et al. [168]
Kaempherol-3-rutinoside-3-rhamnoside	banana peel	740.7	C_33_H_40_O_19_	5.32 ^d^	Rebello et al. [168]
Quercetin-7-rutinoside	banana peel	610.5	C_27_H_30_O_16_	8.78 ^d^	Rebello et al. [168]
Quercetin-3-rutinoside	banana peel	610.5	C_27_H_30_O_16_	29.87 ^d^	Rebello et al. [168]
Kaempferol-7-rutinoside	banana peel	594.52	C_27_H_30_O_15_	4.12 ^d^	Rebello et al. [168]
Laricitrin-3-rutinoside	banana peel	640.16	C_28_H_32_O_17_	2.22 ^d^	Rebello et al. [168]
Kaempferol-3-rutinoside	banana peel	594.52	C_27_H_30_O_15_	12.35 ^d^	Rebello et al. [168]
Isorhamnetin-3-rutinoside	banana peel	624.5	C_28_H_32_O_16_	1.31 ^d^	Rebello et al. [168]
Syringetin-3-rutinoside	banana peel	654.6	C_29_H_34_O_17_	0.63 ^d^	Rebello et al. [168]
Flavonoids—flavanones					
Naringenin	banana peel			84.7 ^f^	Behiry et al. [169]
Flavonoids-flavanols					
Catechin	banana peel	290.27	C_15_H_14_O_6_	1.34 ^d^	Rebello et al. [168]
Epicatechin	banana peel	290.27	C_15_H_14_O_6_	2.55 ^d^	Rebello et al. [168]
Gallocatechin	banana peel	306.27	C_15_H_14_O_7_	4.20 ^d^	Rebello et al. [168]
Procyanidin B1	banana peel	578.14	C_30_H_26_O_12_	1.27 ^d^	Rebello et al. [168]
Procyanidin B2	banana peel	578.14	C_30_H_26_O_12_	81.95 ^d^	Rebello et al. [168]
Procyanidin B4	banana peel	578.14	C_30_H_26_O_12_	7.90 ^d^	Rebello et al. [168]
Other compounds					
Cycloeucalenol acetate	banana pseudostem and rhizome	468.77	C_32_H_52_O_2_		Kandasamy et al. [170]
4-epicyclomusalenone	banana pseudostem and rhizome	424.71	C_30_H_48_O		Kandasamy et al. [170]

^a^ expressed in mg GAE kg^−1^ DM, ^b^ expressed in mg QE kg^−1^ DM, ^c^ depending on the method of extraction, ^d^ expressed in molar proportion (%), ^e^ expressed in ug/mL of crude extract, ^f^ expressed in mg kg^−1^ of dry extract.

**Table 13:** Biological activity and potential applications of phytochemicals obtained from banana residues.

Material	Extract/Compound	Biological Activity/Application	References
Banana peel	extract	- as additives for formulation of bioactive compounds-rich yogurts - antioxidants activity - DPPH• scavenging activity - ABTS+• scavenging activity - *α*-glucosidase inhibitory activity	Kabir et al. [166]
Banana peel	acetonic, ethanoic, and methanolic extracts	- antioxidant activity - antimicrobial activity against *Staphylococcus aureus*, *Pseudomonas aeruginosa*, *Escherichia Coli, Saccharomyces cerevisiae*	Chaudhry et al. [167]
Banana peel	extract	- application as corrosion inhibitors	Vani et al. [176]
Banana pseudostem and rhizome	crude extracts (hexane, chloroform, ethyl acetate, and methanolic) Isolates: chlorogenic acid 4-epicyclomusalenone cycloeucalenol acetate	- antioxidant activity - platelet aggregation inhibitory activity - antimicrobial activity - cytotoxicity	Kandasamy et al. [170]
Banana peel	extract	- antioxidant activity	Rebello et al. [168]
Yellow and red banana peel	hydroalcoholic extracts	- the antioxidant, cytotoxic, and antimicrobial effects	Avram et al. [170]
Banana peel	Methanolic extract	- application as biofungicide against the growth of *Fusarium culmorum* and *Rhizoctonia solani*, and as a bactericide against *Agrobacterium tumefaciens* for natural wood preservation during handling or in service.	Behiry et al. [169]
Banana peel, pulp, seed, and flower	Ethanolic extract	- very strong antioxidant activity - antihyperglycemic activity at a dose of 350 mg/kg body weight	Nofianti et al. [172]
Banana peel	Water extract contained ethanediol and butanediol	- highly reducing agent for metals used for the synthesis of silver nanoparticles	Buendía-Otero et al. [174]
Banana inflorescence		- as good biocolorants with attractive colors, moderate stability in food systems, water-solubility, and benefits for health	Padam et al. [175]

**Table 14:** Total phenolic content (TPC), total flavonoid content (TFC), and main phytochemicals identified and quantified in apple pomace.

Name	MW [g mol^−1^]	C_x_H_y_O_z_	Concentration [mg/kg dm *]	References
Total phenolic content (TPC)			2620–8560 ^a^ 1590–10,620 ^a^ 4399–8100 ^a^	Waldbauer [181] Li et al. [182] Gorjanović et al. [183]
Total flavonoid content (TFC)			18,600–27,400 ^b^	Gorjanović et al. [183]
Phenolic acids—hydroxybenzoic acids				
Gallic acid	170.12	C_7_H_6_O_5_	2.22–4.80 ^d^	Gorjanović et al. [183]
4-hydroxybenzoic acid	137.02	C_7_H_5_O_3_	17.66–69.56 ^c^	Li et al. [182]
Protocatechuic acid	154.12	C_7_H_6_O_4_	2.78–30.50 ^c^	Li et al. [182]
*p*-hudroxybenzoic acid	138.22	C_7_H_6_O_3_	1.16–5.80 ^d^	Gorjanović et al. [183]
Cyclohexanecarboxylic acids				
Quinic acid	192.17	C_7_H_12_O_6_	227.4–418 ^c^	Uyttebroek et al. [179]
Phenolic acids—hydroxycinnamic acids				
Chlorogenic acid	354.31	C_16_H_18_O_9_	41.80 –160.40 ^c^ 89.0–308.3 ^d^ 38.9–312.8 960	Li et al. [182] Gorjanović et al. [183] Uyttebroek et al. [179] Pingret et al. [189]
*p*-coumaroylquinic acid	338.31	C_16_H_18_O_8_	94	Pingret et al. [189]
Sinapic acid	224.212	C_11_H_12_O_5_	2.03–7.20 ^d^	Gorjanović et al. [183]
Caffeic acid	180.16	C_9_H_8_O_4_	0.12–0.35 ^d^	Gorjanović et al. [183]
*p*-Coumaric acid	164.16	C_9_H_8_O_3_	2.52–23.11 ^c^ 0.32–0.76 ^d^	Li et al. [182] Gorjanović et al. [183]
Ferulic acid	194.18	C_10_H_10_O_4_	1.70–4.21 ^c^ 13.24–23.80 ^d^	Li et al. [182] Gorjanović et al. [183]
Flavonoids—flavonols				
Rutin	610.52	C_27_H_30_O_16_	7.99–46.93 ^d^ 19.32 2.24–3.26 ^c^ 10 ^b^	Gorjanović et al. [183] Oleszek et al. [185] Uyttebroek et al. [179] Pingret et al. [189]
Quercetin	302.24	C_15_H_10_O_7_	7.2–14.2 ^d^ 25.2 ^e^	Gorjanović et al. [183] Oleszek et al. [185]
Quercetin-3-*O*-galactoside	464.38	C_21_H_20_O_12_	80.8–165.2 ^d^	Gorjanović et al. [183]
Quercetin-3-*O*-pentosyl	434.35	C_20_H_18_O_11_	44.8 ^e^	Oleszek et al. [185]
Hyperoside	464.38	C_21_H_20_O_12_	434 ^e^ 122 ^b^	Oleszek et al. [185] Pingret et al. [189]
Isoquercetin	464.38	C_21_H_20_O_12_	70 ^e^ 42	Oleszek et al. [185] Pingret et al. [189]
Quercitrin	448.38	C_21_H_20_O_11_	442.4 ^e^ 70.14–109.5 ^c^ 40 ^b^	Oleszek et al. [185] Uyttebroek et al. [179] Pingret et al. [189]
Isoquercitrin	464.0955	C_21_H_20_O_12_	10.65–15.5 ^c^	Uyttebroek et al. [179]
Avicularin	434.35	C_20_H_18_O_11_	285.6 ^e^ 81.6–125.7 24	Oleszek et al. [185] Uyttebroek et al. [179] Pingret et al. [189]
Reynoutrin	434.35	C_20_H_18_O_11_	145.6 ^e^ 54 ^b^	Oleszek et al. [185] Pingret et al. [189]
Isorhamnetin			1.10–17.62 ^d^	Gorjanović et al. [183]
Isorhamnetin-3-*O*-arabinofuranoside	478.41	C_22_H_22_O_12_		Ramirez–Ambrosi et al. [186]
isorhamnetin-3-*O*-pentoside	478.41	C_22_H_22_O_12_		Ramirez–Ambrosi et al. [186]
Isorhamnetin-3-*O*-rutinoside	624.55	C_28_H_32_O_16_	0.10–1.11 ^d^	Gorjanović et al. [183]
Isorhamnetin-3-*O*-rhamnoside	462.41	C_22_H_22_O_11_		Ramirez–Ambrosi et al. [186]
Kaempferol	286.24	C_15_H_10_O_6_	0.62–2.46 ^d^	Gorjanović et al. [183]
Kaempferol-7-*O*-glucoside	448.38	C_21_H_20_O_11_	0.03–1.19 ^d^	Gorjanović et al. [183]
Quercetin-3-*O*-rhamnoside	448.38	C_21_H_20_O_11_	34.1–121.9 ^d^	Gorjanović et al. [183]
Guajavarin	434.353	C_20_H_18_O_11_	161 ^b^	Pingret et al. [189]
Hyperin	463.371	C_21_H_19_O_12_	64.02–92.4 ^c^	Uyttebroek et al. [179]
Flavonoids—flavanonols				
Taxifolin	304.254	C_15_H_12_O_7_	0.16–0.46 ^d^	Gorjanović et al. [183]
Flavonoids—flavanols				
Catechin	290.27	C_15_H_14_O_6_	1.50 –31.70 ^c^ 1.05–7.45 ^c^ 52	Li et al. [182] Uyttebroek et al. [179] Pingret et al. [189]
Epicatechin	290.27	C_15_H_14_O_6_	34.4–166.3 ^c^ 244	Uyttebroek et al. [179] Pingret et al. [189]
Procyanidin	594.53	C_30_H_26_O_13_	2900 3408	Fernandes et al. [178] Pingret et al. [189]
Procyanidin B2	578.52	C_30_H_26_O_12_	42.8–208.1	Uyttebroek et al. [179]
Flavonoids—flavanones				
Naringenin	272.26	C_15_H_12_O_5_	0.11–0.24 ^d^	Gorjanović et al. [183]
Eriodictyol	288.26	C_15_H_12_O_6_	0.11–0.21 ^d^	Gorjanović et al. [183]
Naringin	580.541	C_27_H_32_O_14_	0.22–0.60 ^d^	Gorjanović et al. [183]
Flavonoids—flavones				
Apigenin	270.24	C_15_H_10_O_5_	0.31–0.48 ^d^	Gorjanović et al. [183]
Apigenin-7-*O*-glucoside	432.38	C_21_H_20_O_10_	0.47–1.01 ^d^	Gorjanović et al. [183]
Chrysin	254.25	C_15_H_10_O_4_	0.11–0.22 ^d^	Gorjanović et al. [183]
Luteolin	286.24	C_15_H_10_O_6_	0.10–0.26 ^d^	Gorjanović et al. [183]
Flavonoids—dihydrochalcones				
Phloretin	274.26	C_15_H_14_O_5_	0.29–0.98 ^d^	Gorjanović et al. [183]
Phlorizin	436.4	C_21_H_24_O_10_	112–215 ^d^ 361.2 ^f^ 56.8–198.6 ^c^ 1008	Gorjanović et al. [183] Oleszek et al. [185] Uyttebroek et al. [179] Pingret et al. [189]
Phloretin 2-*O*-glucoside	452.41	C_21_H_24_O_11_		Ramirez–Ambrosi et al. [186]
Phloretin -xylosyl-glucoside	568.52	C_26_H_32_O_14_	142	Pingret et al. [189]
3-hydroxyphloretin-2′-*O*-xylosylglucoside	584.52	C_26_H_32_O_15_		Ramirez–Ambrosi et al. [186]
3-hydroxyphloretin-2′-*O*-glucoside	452	C_21_H_24_O_11_		Ramirez–Ambrosi et al. [186]
Coumarins **				
Aesculin	340.282	C_15_H_16_O_9_	5.53–10.67	Gorjanović et al. [183]
(E)-12-(2′-Chlorovinyl) bergapten	277.5	C_14_H_10_O_4_Cl		Mohammed and Mustafa [187]
12-(1′,1′-dihydroxyethyl) bergapten	276	C_14_H_12_O_6_		Mohammed and Mustafa [187]
12-(2′-chloropropan-2′-yl)-8-hydroxybergapten	308.5	C_15_H_13_O_5_Cl		Mohammed and Mustafa [187]
12-Hydroxy-11-chloromethylbergapten	332.5	C_13_H_9_O_5_Cl		Mohammed and Mustafa [187]
officinalin	220	C_11_H_8_O_5_		Khalil and Mustafa [188]
8-(tert-butyl)officinalin	276	C_15_H_16_O_5_		Khalil and Mustafa [188]
8-Hydroxyofficinalin	236	C_11_H_8_O_6_		Khalil and Mustafa [188]
Officinalin-8-acetic acid	278	C_13_H_10_O_7_		Khalil and Mustafa [188]
8-(2′-hydroxypropan-2′-yl) officinalin	289	C_15_H_16_O_6_		Khalil and Mustafa [188]
Triterpenoids				
*α*-amyrin	426.72	C_30_H_50_O	94.0	Woźniak et al. [190]
*β*-amyrin	426.72	C_30_H_50_O	41.4	Woźniak et al. [190]
Uvaol	442.72	C_30_H_50_O_2_	53.9	Woźniak et al. [190]
Erythtodiol	442.72	C_30_H_50_O_2_	18.0	Woźniak et al. [190]
Ursolic aldehyde	440.70	C_30_H_48_O_2_	73.9	Woźniak et al. [190]
Ursolic acid	456.70	C_30_H_48_O_3_	7125.1	Woźniak et al. [190]
Oleanolic acid	456.70	C_30_H_48_O_3_	1591.4	Woźniak et al. [190]
Pomolic acid	472.70	C_30_H_48_O_4_	870.3	Woźniak et al. [190]
Pigments ***				
all-*trans*-neoxanthin	600.884	C_40_H_56_O_4_	1.14–7.11 ^d^	Delgado–Pelayo [191]
all-*trans*-violaxanthin	600.870	C_40_H_56_O_4_	1.70–18.26 ^d^	Delgado–Pelayo [191]
9-*cis*-violaxanthin	600.870	C_40_H_56_O_4_	0.23–2.37 ^d^	Delgado–Pelayo [191]
9-*cis*-Neoxanthin	600.884	C_40_H_56_O_4_	0.56–21.92 ^d^	Delgado–Pelayo [191]
13-*cis*-violaxanthin	600.884	C_40_H_56_O_4_	0.10–0.29 ^d^	Delgado–Pelayo [191]
all-*trans*-antheraxanthin	584.885	C_40_H_56_O_3_	0.09–0.57 ^d^	Delgado–Pelayo [191]
all-*trans*-zeaxanthin	568.886	C_40_H_56_O_2_	0.08–0.52 ^d^	Delgado–Pelayo [191]
all-*trans*-lutein	568.871	C_40_H_56_O_2_	1.32–61.53 ^d^	Delgado–Pelayo [191]
9-*cis*-lutein	568.871	C_40_H_56_O_2_	0.06–1.61 ^d^	Delgado–Pelayo [191]
13-*cis*-lutein	568.871	C_40_H_56_O_2_	0.10–2.76 ^d^	Delgado–Pelayo [191]
all-*trans*-*β*-carotene	536.8726	C_40_H_56_	1.49–30.31 ^d^	Delgado–Pelayo [191]
Monoestrified xanthophylls			3.01–10.18 ^d^	Delgado–Pelayo [191]
Diesterified xanthophylls			4.93–38.39 ^d^	Delgado–Pelayo [191]
Chlorophyll a	893.509	C_55_H_72_MgN_4_O_5_	18.39–1049.26 ^d^	Delgado–Pelayo [191]
Chlorophyll b	907.492	C_55_H_70_MgN_4_O_6_	4.78–309.86 ^d^	Delgado–Pelayo [191]
Other compounds				
Resveratrol	228.24	C_14_H_12_O_3_	0.16–0.89	Gorjanović et al. [183]
Pterostilbene	256.296	C_16_H_16_O_3_	0.19–0.90	Gorjanović et al. [183]
Pinocembrin	256.25	C_15_H_12_O_4_	0.22–0.39	Gorjanović et al. [183]
Palmitic acid	256.4	C_16_H_32_O_2_	7.25 ^f^	Walia [192]
Linoleic acid	280.45	C_18_H_32_O_2_	43.81 ^f^	Walia [192]
Oleic acid	282.47	C_18_H_34_O_2_	46.50 ^f^	Walia [192]
Stearic acid	284.48	C_18_H_36_O_2_	1.72 ^f^	Walia [192]
Arachidic acid	312.54	C_20_H_40_O_2_	0.72 ^f^	Walia [192]
Pinnatifidanoside D	518	C_24_H_38_O_12_	344.4	Oleszek et al. [185]

* dm—dry matter, ^a^ expressed as mg gallic acid equivalent, ^b^ expressed as quercetin equivalent, ^c^ depending on the methods of extraction or apple pressing, ^d^ depending on apple varieties, ^e^ expressed as rutin equivalent, ^f^ expressed in % of the oil extracted from apple seeds, ** determined in seeds, *** determined in peels.

**Table 15:** Biological activity and potential applications of phytochemicals obtained from apple residues.

Material	Extract/Compound	Biological Activity/Application	References
Apple seeds	coumarins	- antioxidant activity - antitumor activity	Khalil and Mustafa [188]
Apple pomace	phenolic-rich fractions: phloridzin, phloretin, quercitrin, and quercetin as major constituents	- anti-inflammatory, cytotoxic activity, anticancer activity (SiHa, KB, and HT-29 cell lines)	Rana et al. [195]
Apple pomace	crude extract and four fractions	- antioxidant activity - antifungal activity against crop pathogens: *Neosartorya fischeri*, *Fusarium oxysporum*, *Botrytis* sp. *Petriella setifera*	Oleszek et al. [185]
Flour from apple pomace	ethanolic extract	antioxidant, antidiabetic, and antiobesity effects	Gorjanović et al. [183]
Apple pomace	Ursolic acid	antimicrobial, anti-inflammatory, and antitumor activities	Cargnin et al. [196]
Apple peel	ursolic acid	antimalarial activity	Silva et al. [197]
Apple pomace	ethanolic extract: 5-*O*-caffeoylquinic acid as the major compound	- antioxidant and antimicrobial activity (against *Propionibacterium acnes*) - application in dermal formulations	Arraibi et al. [198]
Apple pomace	Extracts (boiling water with 1% acetic acid) and fractions (polyphenols and carbohydrates)	- antioxidant activity - anti-inflammatory activity - application as a food ingredient in yogurt formulation	Fernandes et al. [178]
Apple pomace	phloretin, phloridzin	antioxidant and antibacterial activity (*Staphylococcus aureus*, *Escherichia coli*)	Zhang et al. [199]
Apple pomace	Phloridzin oxidation products (POP)	application as natural yellow pigments in gelled desserts	Haghighi and Rezaei [200]
Apple pomace	Phloridzin oxidation products (POP)	- strong antioxidant activity - application as a yellow pigment	Liu et al. [201]
Apple peel	extract	- application as corrosion inhibitor for carbon steel	Vera et al. [202]

**Table 16:** Phytochemicals identified and quantified in grape residues.

Name	MW [g mol^−1^]	C_x_H_y_O_z_	Concentration [mg/kg dm]	References
Total phenolic content (TPC)			280–7770 ^b,e,f^ 14,200–26,700 ^a,e^	Pintać et al. [208] Eyiz et al. [209]
Total flavonoid content (TFC)			40–1150 ^b,e,f^ 2403–4178 ^a,e^	Pintać et al. [208] Eyiz et al. [209]
Total monomeric anthocyanins			539–1598 ^a,e^	Eyiz et al. [209]
Total proanthocyanidin			3.23–6.32 ^a,e^	Eyiz et al. [209]
Phenolic acids—hydroxybenzoic acid				
Gallic acid	170.12	C_7_H_6_O_5_	24–246 ^a,e^ 250 ^a^ 4.86–70 ^a,e,f^ 75.5 ^a^ 596.36 ^a^ 3030 ^c^	Farías–Campomanes et al. [210] Wang et al. [211] Pintać et al. [208] Daniel et al. [212] Wittenauer et al. [213] Jara-Palacios et al. [214]
Digalloylquinic acid	496.4	C_21_H_20_O_14_	299 ^a^	Gonçalves et al. [215]
Ellagic acid	302.197	C_14_H_6_O_8_	620 ^a^ 8.37–64.1 ^b,e,f^ 4.315 ^a^	Wang et al. [211] Pintać et al. [208] Daniel et al. [212]
Protocatechuic acid	154.12	C_7_H_6_O_4_	9–63 ^a,e^ 940 ^c^	Farías–Campomanes et al. [210] Jara–Palacios et al. [214]
Vanillic acid	168.15	C_8_H_8_O_4_	24–237 ^a,e^ 0.53–13.0 ^b,e,f^ 10 ^a^	Farías–Campomanes et al. [210] Pintać et al. [208] Daniel et al. [212]
4-hydroxybenzoic acid	138.122	C_7_H_6_O_3_	9–63 ^a,e^ 0.16–1.71 ^b,e,f^	Farías–Campomanes et al. [210] Pintać et al. [208]
Syringic acid	198.17	C_9_H_10_O_5_	48–593 ^a,e^ 0.13–20.6 ^b,e,f^	Farías–Campomanes et al. [210] Pintać et al. [208]
Galloylshikimic acid	326.25	C_14_H_14_O_9_	438.1 ^a^	Gonçalves et al. [215]
Phenolic acids—hydroxycinnamic acid				
Chlorogenic acid	354.31	C_16_H_18_O_9_	0.14–11.50 ^b,e,f^ 4.715 ^a^	Pintać et al. [208] Daniel et al. [212]
Caffeic acid	180.16	C_9_H_8_O_4_	0.41–1.68 ^b,e,f^ 9.735 ^a^ 630 ^c^	Pintać et al. [208] Daniel et al. [212] Jara–Palacios et al. [214]
Caftaric acid	312.23	C_13_H_12_O_9_	735.32 ^a^ 880 ^c^ 11–168 ^a,g^	Wittenauer et al. [213] Jara–Palacios et al. [214] Jara–Palacios et al. [216]
cis-Coutaric acid	296.23	C_13_H_12_O_8_	5.3–11.8 ^a,g^	Jara–Palacios et al. [216]
trans-coutaric	296.23	C_13_H_12_O_8_	5.5–20.7 ^a,g^	Jara–Palacios et al. [216]
*p*-Coumaric acid	164.16	C_9_H_8_O_3_	6–39 ^a,e^ 0.13–1.49 ^b,e,f^ 8.175 ^a^ 510 ^c^	Farías–Campomanes et al. [210] Pintać et al. [208] Daniel et al. [212] Jara–Palacios et al. [214]
Flavonoids—flavonols				
Quercetin	302.236	C_15_H_10_O_7_	3–15 ^a,e^ 11.3–78.9 ^b,e,f^ 200 ^a^ 2.473–15.637 ^c^ 4.7 ^a^ 2870 ^c^ 344–403 ^c,f^	Farías–Campomanes et al. [210] Pintać et al. [208] Wang et al. [211] Balea et al. [217] Daniel et al. [212] Jara–Palacios et al. [214] Drosou et al. [218]
Quercetin-3-*O*-glucoside	463.371	C_21_H_19_O_12_	0.39–38.0 ^b,e,f^ 67.6 ^a^ 2374.32 ^a^ 16,900 ^c^ 475–609 ^c,f^	Pintać et al. [208] Gonçalves et al. [215] Wittenauer et al. [213] Jara–Palacios et al. [214] Drosou et al. [218]
Quercetin-3-*O*-glucuronide	478.362	C_21_H_18_O_13_	13.4 ^a^ 2432.29 ^a^ 15,800 ^c^ 990–1285 ^c,f^	Gonçalves et al. [215] Wittenauer et al. [213] Jara–Palacios et al. [214] Drosou et al. [218]
Quercetin-3-*O*-pentoside	434.35	C_20_H_18_O_11_	52.0 ^a^	Gonçalves et al. [215]
Quercetin-3-*O*-rhamnoside	448.4	C_21_H_20_O_11_	49.4 ^a^	Gonçalves et al. [215]
Quercetin-3-*O*-galactoside			2120 ^c^	Jara–Palacios et al. [214]
Hyperoside	464.38	C_21_H_20_O_12_	0.17–5.67 ^b,e,f^	Pintać et al. [208]
Rutin	610.52	C_27_H_30_O_16_	0.11–8.19 ^b,e,f^ 2.136 ^c^ 5.3 ^a^ 690 ^c^	Pintać et al. [208] Balea et al. [217] Daniel et al. [212] Jara–Palacios et al. [214]
Isorhamnetin	316.265	C_16_H_12_O_7_	6.42–72.9 ^b,e,f^	Pintać et al. [208]
Isorhamnetin 3-*O*-glucoside	478.406	C_22_H_22_O_12_	66.3 ^a^ 145–175 ^c,f^	Gonçalves et al. [215] Drosou et al. [218]
Myricetin	318.24	C_15_H_10_O_8_	170 ^a^ 0.21–2.31 ^b,e,f^ 0.341–1.029 ^c^ 452–711 ^c,f^	Wang et al. [211] Pintać et al. [208] Balea et al. [217] Drosou et al. [218]
Myricetin-3-*O*-hexoside	480.38	C_21_H_20_O_13_	184.6 ^a^	Gonçalves et al. [215]
Myricetin-3-*O*-glucoside	480.38	C_21_H_20_O_13_	781–1044 ^c^	Drosou et al. [218]
Quercitrin	448.38	C_21_H_20_O_11_	0.21–3.99 ^b,e,f^	Pintać et al. [208]
Laricitrin-*O*-hexoside	494.405	C_22_H_22_O_13_	46.8 ^a^ 216–434 ^c,f^	Gonçalves et al. [215] Drosou et al. [218]
Kaemferol	286.239	C_15_H_10_O_6_	80 ^a^ 2.45–53.1 ^b,e,f^ 3.38–5.74 ^c^ 150 ^c^	Wang et al. [211] Pintać et al. [208] Balea et al. [217] Jara–Palacios et al. [214]
Kaempferol 3-*O*-glucoside	448.38	C_21_H_20_O_11_	0.05–23.0 ^b,e,f^ 3670 ^c^	Pintać et al. [208] Jara–Palacios et al. [214]
Kaempferol 3-glucuronide	462.4	C_21_H_18_O_12_	310 ^c^	Jara–Palacios et al. [214]
Syringetin 3-glucoside	508.432	C_23_H_24_O_13_	168–200 ^c,f^	Drosou et al. [218]
Quercitrin	448.38	C_21_H_20_O_11_	3.272–14.952 ^c^	Balea et al. [217]
Isoquercitrin	464.0955	C_21_H_20_O_12_	2.429–65.698 ^c^	Balea et al. [217]
Flavonoids—flavanols				
Catechin	290.27	C_15_H_14_O_6_	1460 ^a^ 5.01–193 ^b,e,f^ 945 ^a^ 1101.7 ^a^ 10,496.63 ^a^ 12,200 ^c^	Wang et al. [211] Pintać et al. [208] Gonçalves et al. [215] Daniel et al. [212] Wittenauer et al. [213] Jara–Palacios et al. [214]
Epicatechin	290.271	C_15_H_14_O_6_	1280 ^a^ 5.80–309 ^b,e,f^ 949 ^a^ 322.5 ^a^ 8994.93 ^a^ 6340 ^c^	Wang et al. [211] Pintać et al. [208] Gonçalves et al. [215] Daniel et al. [212] Wittenauer et al. [213] Jara–Palacios et al. [214]
Epigallocatechin	306.27	C_15_H_14_O_7_	900 ^a^	Wang et al. [211]
Procyanidin dimers	578.1424	C_30_H_26_O_12_	3306 ^a^	Gonçalves et al. [215]
Procyanidin trimers	866.77	C_45_H_38_O_18_	1105 ^a^ 12,920 ^c^	Gonçalves et al. [215] Jara–Palacios et al. [214]
Procyanidin tetramer	1155.0	C_60_H_50_O_24_	806 ^a^ 16,540 ^c^	Gonçalves et al. [215] Jara–Palacios et al. [214]
Procyanidin B1	578.1424	C_30_H_26_O_12_	4858.58 ^c^ 15,500 ^c^	Wittenauer et al. [213] Jara–Palacios et al. [214]
Procyanidin B2	578.1424	C_30_H_26_O_12_	4277.04 ^c^ 4940 ^c^	Wittenauer et al. [213] Jara–Palacios et al. [214]
Procyanidin B3	578.1424	C_30_H_26_O_12_	4350 ^c^	Jara–Palacios et al. [214]
Procyanidin B4	578.1424	C_30_H_26_O_12_		Jara–Palacios et al. [216]
Flavonoids—flavones				
Apigenin	270.24	C_15_H_10_O_5_	0.58 ^b^	Pintać et al. [208]
Apigenin 7-*O*-glucoside	432.38	C_21_H_20_O_10_	0.02–12.7 ^b,e,f^	Pintać et al. [208]
Luteolin	286.24	C_15_H_10_O_6_	0.23–1.07 ^b,e,f^	Pintać et al. [208]
Luteolin-7-*O*-glucoside	448.38	C_21_H_20_O_11_	0.36–4.46 ^b,e,f^	Pintać et al. [208]
Flavonoids—flavanones				
Chrysoeriol	300.27	C_16_H_12_O_6_	0.04–0.51 ^b,e,f^	Pintać et al. [208]
Naringenin	272.26	C_15_H_12_O_5_	0.11–0.83 ^b,e,f^	Pintać et al. [208]
Flavonoids-flavanonols				
Astilbin	450.396	C_21_H_22_O_11_	3120–4200 ^b,e^	Negro et al. [219]
Flavonoids—anthocyanins				
Delphinidin 3-*O*-glucoside	465.387	C_21_H_21_O_12_	4.68–54.7 ^b,e,f^ 775–936 ^c,f^ 7–57 ^a,e^	Pintać et al. [208] Drosou et al. [218] Negro et al. [219]
Cyanidin 3-*O*-glucoside	449.388	C_21_H_21_O_11_	2.21–11.3 ^b,e,f^ 3–37 ^b,e^	Pintać et al. [208] Negro et al. [219]
Petunidin-3-*O*-glucoside	479.41	C_22_H_23_O_12_	1.28–35.4 ^b,e,f^ 77.0 ^a^ 1295–1618 ^c,f^	Pintać et al. [208] Gonçalves et al. [215] Drosou et al. [218]
Peonidin-3-*O*-glucoside	463.41	C_22_H_23_O_11_	1.51–64.7 ^b,e,f^ 202.2 ^a^ 1591–2044 ^c,f^	Pintać et al. [208] Gonçalves et al. [215] Drosou et al. [218]
Malvidin 3-glucoside	493.441	C_23_H_25_O_12_	0.80–384 ^b,e,f^ 443.0 ^a^ 12,182–17,687 ^c,f^	Pintać et al. [208] Gonçalves et al. [215] Drosou et al. [218]
Peonidin-3-*O*-acetyl glucoside	505.4	C_24_H_25_O_12_^+^	90.2 ^a^	Gonçalves et al. [215]
Malvidin 3-*O*-acetyl glucoside	535.5	C_25_H_27_O_13_^+^	96.2 ^a^ 937–1182 ^c,f^	Gonçalves et al. [215] Drosou et al. [218]
Malvidin 3-caffeoyl glucoside	655.6	C_32_H_31_O_15_	1079–1450 ^c,f^	Drosou et al. [218]
Petunidin 3-coumaroyl glucoside	625.5536	C_31_H_29_O_14_	735–806 ^c,f^	Drosou et al. [218]
Peonidin 3-coumaroyl glucoside	609.5542	C_31_H_29_O_13_	796–1231 ^c,f^	Drosou et al. [218]
Malvidin-3-coumaroyl glucoside	639.58	C_32_H_31_O_14_	4700–7232 ^c,f^	Drosou et al. [218]
Delphinidin	303.24	C_15_H_11_O_7_	5570 ^a^	Wang et al. [211]
Cyanidin	287.24	C_15_H_11_O_6_	3620 ^a^	Wang et al. [211]
Petunidin	317.27	C_16_H_13_O_7_	15,500 ^a^	Wang et al. [211]
Peonidin	301.27	C_16_H_13_O_6_	25,320 ^a^	Wang et al. [211]
Malvidin	331.30	C_17_H_15_O_7_	10,390 ^a^	Wang et al. [211]
Terpenoids				
Ursolic acid	456.70	C_30_H_48_O_3_	0.96–606 ^b,e,f^	Pintać et al. [208]
Coumarins				
Esculetin	178.14	C_9_H_6_O_4_	0.23–0.66 ^b,e,f^	Pintać et al. [208]
Stilbenes				
resveratrol	228.243	C_14_H_12_O_3_	0.07–3.37 ^b,e,f^ 5.3–6.2 ^a,e^	Pintać et al. [208] Iora et al. [220]
Fatty acids				
Palmitic acid (16:1)	256.4	C_16_H_32_O_2_	85.43–110.97 ^d^	Iora et al. [220]
Palmitoleic acid (16:1 n-7)	254.414	C_16_H_30_O_2_	7.04–13.21 ^d^	Iora et al. [220]
Stearic acid (18:0)	284.48	C_18_H_36_O_2_	26.75–38.77 ^d^	Iora et al. [220]
Oleic acid (18:1 n-9)	282.47	C_18_H_34_O_2_	118.15–141.54 ^d^	Iora et al. [220]
Linoleic acid (18:2 n-6)	280.4472	C_18_H_32_O_2_	627.21–684.47 ^d^	Iora et al. [220]
Linolenic acid (18:3 n-3)	278.43	C_18_H_30_O_2_	11.26–19.97 ^d^	Iora et al. [220]
Arachidic acid (20:0)	312.5304	C_20_H_40_O_2_	3.12–3.45 ^d^	Iora et al. [220]
Eicosenoic acid 20:1 n-9	310.51	C_20_H_38_O_2_	0.89–2.57 ^d^	Iora et al. [220]
Behenic acid 22:0	340.58	C_22_H_44_O_2_	1.47–2.42 ^d^	Iora et al. [220]
Lignoceric acid 24:0	368.63	C_24_H_48_O_2_	1.03–1.67 ^d^	Iora et al. [220]
SFA			117.79–157.07 ^d^	Iora et al. [220]
MUFA			131.56–156.95 ^d^	Iora et al. [220]
PUFA			647.17–695.73 ^d^	Iora et al. [220]
n-6/n-3			31.43–60.80 ^d^	Iora et al. [220]
SFA/PUFA			0.17–0.24 ^d^	Iora et al. [220]
TFA			938.41–945.08 ^d^	Iora et al. [220]
Other compounds				
Vanillin	152.15	C_8_H_8_O_3_	25.5 ^a^	Daniel et al. [212]
trans-piceid	390.388	C_20_H_22_O_8_	7.75 ^a^	Daniel et al. [212]

^a^ expressed in mg per kg of dry matter (DM), ^b^ expressed in mg per kg of fresh weight, ^c^ expressed in mg per kg of the extract, ^d^ expressed in mg per g of total lipids extracted from grape pomace, ^e^ depending on methods of extraction, ^f^ depending on varieties of grapes, ^g^ depending on the part of the pomace: seeds, skins, stems.

**Table 17:** Biological activity and potential applications of phytochemicals obtained from grape residues.

Material	Extract/Compound	Biological Activity/Application	References
Fresh and fermented grape pomace	Extract	- antioxidant, anti-inflammatory, and antiproliferative activity	Balea et al. [217]
Grape pomace	Hydroalcoholic extract (saponins, tannins, and flavonoids as active constituents)	- anthelmintic activity	Soares et al. [229]
Grape pomace	Whole apple pomace (phenolic compounds as main constituents)	- reduction of the severity of non-alcoholic hepatic steatosis - inhibition of steatohepatitis - improvement in insulin sensitivity - reduction of ectopic fat deposition in mice	Daniel et al. [212]
Grape pomace	crude extract and four fractions: the most active free phenolic acids fraction	- inhibitory effect on collagenase and elastase	Wittenauer et al. [213]
White grape pomace	extract: catechin, epicatechin, quercetin, and gallic acid as the main active constituents	- antiproliferative activity against adenocarcinoma cell	Jara–Palacios et al. [214]
Grape pomace	Ethanolic extract	- antioxidant activity - potential application as additives to food enhancing nutritional value and improving storability	Iora et al. [220]
Grape stem	extracts	- prevention of radical oxidation of the polyunsaturated fatty acids of low-density lipoproteins (LDL) - reduction of intracellular reactive oxygen species (ROS) - prevention of cardiovascular diseases	Anastasiadi et al. [223]
Grape seeds	procyanidin-rich extract	- antibacterial activity against *Helicobacter pylori* (H. pylori)	Silvan et al. [230]
Grape seeds	procyanidin-rich extract	- antihypertensive activity	Quiñones et al. [231]
Grape pomace	phenolics	- antioxidant properties	Tournour et al. [232]
Grape pomace	“Enocianina”—anthocyanin-rich extract	- radical scavenging, enzymatic, antioxidant and anti-inflammatory activity - application as a colorant in the food industry	Della Vedova et al. [233]
Grape pomace	phenolics	- photoprotective activity - reduction of the negative effects of UV radiation on the skin, such as erythema and photoaging	Hübner et al. [234]
Grape pomace	extracts	- wastewater remediation	Gavrilas et al. [235]
Grape pomace	ethanolic extract	- application as additives to yogurt	Olt et al. [236]
Grape pomace	alcoholic extract	- application as a reducing agent of the precursor silver nitrate, a process that has led to the obtaining of silver nanoparticles (NP Ag) by reducing the ions.	Asmat–Campos et al. [237]
Grape skin	resveratrol	- as an antioxidant in the meat industry	Andrés et al. [238]
Grape seeds	flavonoids	- antimicrobial activity in meat	Biniari et al. [239]
Grape steam	procyanidins	- inhibition of toxic compounds	Bordiga et al. [240]
Grape pulp	phenolic compounds	- pigment protection in meat	Chen et al. [241]
Grape pomace	anthocyanins	- modulation of the sensory characteristic of meat	Crupi et al. [242]
Grape pomace	stilbenes	- modulation of the sensory characteristic of meat	Mainente et al. [243]
Grape seeds	Unsaturated fatty acids (linoleic and oleic acid)	- substitution nitrate and nitrite	Gárcia–Lomillo and González-San José [244]

**Table 18:** Phytochemicals identified and quantified in citrus residues.

Name	Citrus Residues	MW [g mol^−1^]	C_x_H_y_O_z_	Concentration [mg/kg dm]	References
Total phenols	kinnow peel			13,840–27,910 ^a,c^	Yaqoob et al. [246]
	lime peel			5.2 ^b^	Karetha et al. [247]
	mandarin peel			4.0 ^b^	Karetha et al. [247]
	lemon peel			4.7 ^b^	Karetha et al. [247]
	pomelo peel			6.4 ^b^	Karetha et al. [247]
	rough lemon peel			4.1 ^b^	Karetha et al. [247]
	citron peel			6.8 ^b^	Karetha et al. [247]
	sour orange peel			30.4–1354.4 ^a^	Benayad et al. [248]
	lime and orange peel			3860	Barbosa et al. [249]
	orange peel			7055–19,885 ^a^	Liew et al. [250]
	orange seeds oil			4430	Jorge et al. [251]
Total flavonoids	kinnow peel			610–11,770 ^a^	Yaqoob et al. [246]
	sour orange peel			2.3–603.6 ^a^	Benayad et al. [248]
	orange peel			854.7–2975.4 ^a^	Liew et al. [250]
	sour orange peel			589.4	Olfa et al. [252]
	lime peel			95.3	Olfa et al. [252]
	orange peel			132.2	Olfa et al. [252]
	lemon peel			610.5	Olfa et al. [252]
	mandarin peel			275.9	Olfa et al. [252]
Total carotenoids	orange seeds oil			19	Jorge et al. [251]
Organic acids					
Lactic acid	orange peel	90.08	C_3_H_6_O_3_	5463–9861 ^a^	Liew et al. [250]
Citric acid	orange peel	192.1	C_6_H_8_O_7_	19,587–27,910 ^a^	Liew et al. [250]
L-mallic acid	orange peel	134.1	C_4_H_6_O_5_	3056–5064 ^a^	Liew et al. [250]
Kojic acid	orange peel	141.1	C_6_H_6_O_4_	111.2–116.4 ^a^	Liew et al. [250]
Ascorbic acid	orange peel	176.1	C_6_H_8_O_6_	1.12–7.32 ^a^	Liew et al. [250]
Phenolic acids—hydroxybenzoic acids					
Ellagic acid	lime and orange peel	302.20	C_14_H_6_O_8_	109.7	Barbosa et al. [249]
Gallic acid	lime and orange peel sour orange peel orange peel	170.12	C_7_H_6_O_5_	5.7 111.3–866.7 ^a^ 8.84–17.81 ^a^	Barbosa et al. [249] Benayad et al. [249] Liew et al. [250]
Protocatechuic acid	orange peel	154.12	C_7_H_6_O_4_	24.55–65.92 ^a^	Liew et al. [250]
4-hydroxybenzoic acid	orange peel	138.12	C_7_H_6_O_3_	26.27–42.50 ^a^	Liew et al. [250]
Phenolic acids—hydroxycinnamic acids					
Ferulic acid	sour orange peel orange peel yuzu peel sour orange peel mandarin peel lime peel grapefruit peel lemon peel orange peel	194.18	C_10_H_10_O_4_	360.0–17,237.7 ^a^ 154.8–477.3 ^a^ 135 139 101 18 29 18 19	Benayad et al. [248] Liew et al. [250] Lee et al. [253] Lee et al. [253] Lee et al. [253] Lee et al. [253] Lee et al. [253] Lee et al. [253] Lee et al. [253]
*p*-coumaric acid	sour orange peel yuzu peel sour orange peel mandarin peel lime peel grapefruit peel lemon peel orange peel	164.16	C_9_H_8_O_3_	242.4 101 123 52 76 16 48 18	Benayad et al. [248] Lee et al. [253] Lee et al. [253] Lee et al. [253] Lee et al. [253] Lee et al. [253] Lee et al. [253] Lee et al. [253]
Chlorogenic acid	mandarin peel sour orange peel yuzu peel sour orange peel mandarin peel	354.31	C_16_H_18_O_9_	0.08–68.58 ^a^ 4.494 39 96 40	Šafranko et al. [254] Benayad et al. [248] Lee et al. [253] Lee et al. [253] Lee et al. [253]
Caffeic acid	sour orange peel orange peel yuzu peel sour orange peel mandarin peel lime peel lemon peel	180.16	C_9_H_8_O_4_	384.0–1326.1 ^a^ 54.5–210.1 ^a^ 55 27 15 4 12	Benayad et al. [248] Liew et al. [250] Lee et al. [253] Lee et al. [253] Lee et al. [253] Lee et al. [253] Lee et al. [253]
Flavonoids—flavonols					
Rutin	mandarin peel orange peel mandarin peel	610.52	C_27_H_30_O_16_	0.18–4.27 ^a^ 9.56–10.11 ^a^ 177	Šafranko et al. [254] Liew et al. [250] Lee et al. [253]
Flavonoids—flavanols					
Catechin	sour orange peel orange peel	290.26	C_15_H_14_O_6_	378.3–1296 ^a^ 40.92–366.8 ^a^	Benayad et al. [248] Liew et al. [250]
Epigallocatechin	orange peel			84.23–317.14 ^a^	Liew et al. [250]
Flavonoids-flavones					
Apigenin	sour orange peel orange peel	270.24	C_15_H_10_O_5_	38,552.1 57.91–159.67	Benayad et al. [248] Liew et al. [250]
Diosmetin	lime and orange peel	300.26	C_16_H_12_O_6_	3.2	Barbosa et al. [249]
Vitexin	orange peel	432.38	C_21_H_20_O_10_	30.73–117.27 ^a^	Liew et al. [250]
Luteolin	orange peel	286.24	C_15_H_10_O_6_	93.47–275.14 ^a^	Liew et al. [250]
Tangeretin	lime and orange peel	372.37	C_20_H_20_O_7_	14.1	Barbosa et al. [249]
Flavonoids-flavanones					
Naringenin	lime and orange peel sour orange peel	272.25	C_15_H_12_O_5_	4.7 5745.6–96,942 ^a^	Barbosa et al. [249] Benayad et al. [248]
Hesperetin	lime and orange peel	302.28	C_16_H_14_O_6_	10.5	Barbosa et al. [249]
Hesperidin	lime and orange peel mandarin peel yuzu peel mandarin peel lime peel lemon peel orange peel	610.57	C_28_H_34_O_15_	2326.5 0.16–15.07 ^a^ 5367 21,496 4862 6400 16,299	Barbosa et al. [249] Šafranko et al. [254] Lee et al. [253] Lee et al. [253] Lee et al. [253] Lee et al. [253] Lee et al. [253]
Naringin	lime and orange peel yuzu peel sour orange peel mandarin peel lime peel grapefruit peel lemon peel	580.54	C_27_H_32_O_14_	10.2 5255 19,750 146 36 31,314 41	Barbosa et al. [249] Lee et al. [253] Lee et al. [253] Lee et al. [253] Lee et al. [253] Lee et al. [253] Lee et al. [253]
Narirutin	lime and orange peel mandarin peel yuzu peel sour orange peel mandarin peel lime peel grapefruit peel lemon peel orange peel	580.54	C_27_H_32_O_14_	293.4 0.03–5.11 ^a^ 4734 64 10,642 559 2827 185 1342	Barbosa et al. [249] Šafranko et al. [254] Lee et al. [253] Lee et al. [253] Lee et al. [253] Lee et al. [253] Lee et al. [253] Lee et al. [253] Lee et al. [253]
Furanocumarins					
Bergapten	sour orange peel lime peel lemon peel	216.19	C_12_H_8_O_4_	64 196 3	Lee et al. [253] Lee et al. [253] Lee et al. [253]
Bergamottin	lime peel grapefruit peel lemon peel	338.40	C_21_H_22_O_4_	81 25 16	Lee et al. [253] Lee et al. [253] Lee et al. [253]
Volatile compounds					
Caprylaldehyde	sour orange peel	128.21	C_8_H_16_O	180.5 ^b^	Benayad et al. [248]
Decanal	sour orange peel	156.27	C_10_H_20_O	167.2 ^b^	Benayad et al. [248]
Decanol	sour orange peel	158.28	C_10_H_22_O	129.8 ^b^	Benayad et al. [248]
Geranyl Acetate	sour orange peel	196.29	C_12_H_20_O_2_	172.7 ^b^	Benayad et al. [248]
D-limonene	sour orange peel	136.24	C_10_H_16_	3939.4 ^b^	Benayad et al. [248]
*β*-linalool	sour orange peel	154.25	C_10_H_18_O	2038.7 ^b^	Benayad et al. [248]
Linalool oxide	sour orange peel	170.25	C_10_H_18_O_2_	282.0 ^b^	Benayad et al. [248]
Linalyl acetate	sour orange peel	196.29	C_12_H_20_O_2_	589.1 ^b^	Benayad et al. [248]
*β*-myrcene	sour orange peel	136.23	C_10_H_16_	1972.8 ^b^	Benayad et al. [248]
Nerol	sour orange peel	154.25	C_10_H_18_O	106.2 ^b^	Benayad et al. [248]
*β*-ocimene	sour orange peel	136.23	C_10_H_16_	465.2 ^b^	Benayad et al. [248]
*α*-pinene	sour orange peel	136.23	C_10_H_16_	350.1 ^b^	Benayad et al. [248]
*β*-pinene	sour orange peel	136.23	C_10_H_16_	417.6 ^b^	Benayad et al. [248]
*α*-terpineol	sour orange peel	154.25	C_10_H_18_O	389.5 ^b^	Benayad et al. [248]
Carotenoids					
Violaxantin dilaurate	mandarin peel	965.44	C_64_H_100_O_6_	1.33	Huang et al. [255]
Violaxanthin dipalmitate	mandarin peel	1077.7	C_72_H_116_O_6_	2.07	Huang et al. [255]
Zeaxanthin	mandarin peel	568.88	C_40_H_56_O_2_	1.31	Huang et al. [255]
*α*-cryptoxanthin	mandarin peel	552.85	C_40_H_56_O	0.10	Huang et al. [255]
*β*-cryptoxanthin	mandarin peel	552.85	C_40_H_56_O	4.96	Huang et al. [255]
Lutein	kinnow peel mandarin peel	568.87	C_40_H_56_O_2_	9.26–28.89 ^a^ 0.88	Saini et al. [256] Huang et al. [255]
*β*-carotene	mandarin peel	536.87	C_40_H_56_	5.87	Huang et al. [255]
(E/Z)-phytoene	mandarin peel	544.94	C_40_H_64_	25.07	Huang et al. [255]
*β*-citraurin	mandarin peel	432.6	C_30_H_40_O_2_	1.57	Huang et al. [255]
Other compounds					
*α*-tocopherol	orange seeds oil	430.71	C_29_H_50_O_2_	135.7	Jorge et al. [251]
phytosterol	orange seeds oil	414.72	C_29_H_50_O	1304.2	Jorge et al. [251]
malic acid	sour orange peel	134.09	C_4_H_6_O_5_	122.4–2247 ^a^	Benayad et al. [248]

^a^ depending on methods of extraction, ^b^ expressed in mg kg^−1^ of fresh matter of peel, ^c^ expressed in mg kg of the extract.

**Table 19:** Biological activity and potential applications of phytochemicals obtained from citrus residues.

Material	Extract/Compound	Biological Activity/Application	References
sour orange peel	acetone extract chloroform extract ethanol-water extract naringenin gallic acid	- hypoglycaemic and antidiabetic actions - *α*-glucosidase inhibition - *α*-amylase inhibition	Benayad et al. [248]
orange peel	ethanol and methanol extract	- antimicrobial activity against *Xanthomonas, Bacillus subtilis, Azotobacter, Pseudomonas*, *Klebsiella*	Gunwantrao et al. [267]
pomelo peel	extract	- antimicrobial and antioxidants activity	Khan et al. [268]
lemon peel	eriodictoyl, quercetin, and diosmetin	- antiviral activity against SARS-CoV-2	Khan et al. [269]
orange peel	extracts: methanol/water, ethanol/water and acetone/water	- antioxidant activity	Liew et al. [250]
sour orange lime orange lemon mandarin	ethanol/water extracts	- antioxidant activity	Olfa et al. [252]
kinnow peel and pomace	extract (supercritical CO_2_ extraction)	- antioxidant activity - for making functional cookies	Yaqoob et al. [246]
citrus pomace (Persian lime and orange)	extract rich in aglycones of flavanones, mainly naringenin and hesperetin	- activity against *Salmonella enterica* subsp. enterica serovar Typhimurium	Barbosa et al. [265]
lemon, orange andgrapefruit peel	essential oils (EOs)	- antifungal activity against *Rhizoctonia solanii* and *Sclerotium rolfsii* - insecticidal activity against *Rhyzopertha dominica*, *Oryzaephilus* sp., and *Sitophilus granarius*	Achimón et al. [270]
mandarin peel	Extract rich in polyphenols, mainly narirutin and hesperidin	- inhibition of the growth of *Aspergillus flavus*	Liu et al. [271]
citrus peel	nobiletin	- activity against pancreatic cancer through cell cycle arrest	Jiang et al. [272]
citrus peel	nobiletin	- activity against prostate cancer thanks to its anti-inflammation properties	Ozkan et al. [273]
mandarin peel	polymethoxyflavone-rich extract (PMFE)	- alleviating the metabolic syndrome by regulating the gut microbiome and amino acid metabolism	Zeng et al. [263]
Mandarin peel	polymethoxyflavone-rich extract (PMFE)	- alleviating high-fat diet-induced hyperlipidemia	Gao et al. [262]
Orange and lemon peel	Extract rich in flavanones	- reduction in glucose, cholesterol and triglycerides levels in the blood, with positive effects on the regulation of hyperglycemia and lipid metabolism	Chiechio et al. [264]
Lime and orange peel	Extract rich in flavanones, mainly hesperetin, hesperidin, narirutin, and naringin	- antibacterial activity against *Salmonella enterica*	Barbosa et al. [265]
Bitter orange peel	Extract rich in luteolin 7-*O* glucoside	- antioxidant activity - activity against gram-positive bacteria and *Fusarium oxysporum*	Lamine et al. [266]
Mandarin peel	Extract rich in rutin	- activity against gram-negative bacteria and the three pathogenesis fungi: *Bacillus subtilis, Candida albicans and Aspergillus flavus.*	Lamine et al. [266]
Orange peel	Extract rich in polymethoxyflavones	- antifungal activity against *Aspergillus niger*.	Lamine et al. [266]
Pomegranate peel	Ethanolic and methanolic extract	- activity against gram-positive, gram-negative, and two fungal pathogenic strains - used as a natural food preserver	Hanafy et al. [274]

**Table 20:** Phytochemicals identified and quantified in olive waste.

Name	Olive Residue	MW [g mol^−1^]	C_x_H_y_O_z_	Concentration	References
Phenolic acids					
Cinnamic acid	deffated olives	148.16	C_9_H_8_O_2_	2.3 ^a^ 12–205 ^b,c^	Alu’datt et al. [281] Zhao et al. [282]
*p*-coumaric acid	deffated olives olive pomace	164.04	C_9_H_8_O_3_	10.3 ^a^ 84–884 ^b,c^ 5.01 ^b^	Alu’datt et al. [281] Zhao et al. [282] Benincasa et al. [283]
o-coumaric acid	olive pomace	164.04	C_9_H_8_O_3_	70–1562 ^b,c^	Zhao et al. [282]
Caffeic acid	deffated olives leaves OMWW * olive pomace	180.16	C_9_H_8_O_4_	3.1 ^a^ 150 ^b^ 270 ^b^ 39–420 ^b,c^	Alu’datt et al. [281] Ladhari et al. [284] Ladhari et al. [284] Zhao et al. [282]
Protocatechuic acid	deffated olives	154.12	C_7_H_6_O_4_	22.2 ^a^	Alu’datt et al. [281]
Hydroxybenzoic acid	deffated olives	138.12	C_7_H_6_O_3_	4.2 ^a^	Alu’datt et al. [281]
Vanillic acid	deffated olives olive pomace	168.14	C_8_H_8_O_4_	9.0 ^a^ 203–2530 ^b,c^	Alu’datt et al. [281] Zhao et al. [282]
Ferulic acid	deffated olives olive pomace	194.18	C_10_H_10_O_4_	6.9 ^a^ 23–326 ^b,c^	Alu’datt et al. [281] Zhao et al. [282]
Gallic acid	deffated olives olive pomace	170.12	C_7_H_6_O_5_	7.1 ^a^ 7–223 ^b,c^	Alu’datt et al. [281] Zhao et al. [282]
Syringic acid	deffated olives	198.17	C_9_H_10_O_5_	4.1 ^a^	Alu’datt et al. [281]
Sinapic acid	deffated olives	224.21	C_11_H_12_O_5_	14.4 ^a^	Alu’datt et al. [281]
4-hydroxyphenyl acetic acid	olive pomace	152.15	C_8_H_8_O_3_	660–4450 ^b,c^	Zhao et al. [282]
Secoiridoids and derivatives					
Oleuropein	leaves OMWW OMWW olive pomace	540.54	C_25_H_32_O_13_	13,050 ^b^ 9 ^b^ 103 ^b^ 811–12,231 ^b,c^	Ladhari et al. [284] Benincasa et al. [283] Zhao et al. [282]
Oleuropein aglycone	leaves OMWW	378.4	C_19_H_22_O_8_	3410 ^b^ 6 ^b^	Ladhari et al. [284]
Verbascoside	leaves OMWW OMSW ** olive pomace	624.59	C_29_H_36_O_15_	1160 ^b^ 6 ^b^ 5 ^b^ 833–10,159 ^b,c^ 700 ^b^	Ladhari et al. [284] Zhao et al. [282] Benincasa et al. [283]
Ligstroside	leaves OMWW OMSW	524.51	C_25_H_32_O_12_	360 ^b^ 21 ^b^ 56 ^b^	Ladhari et al. [284]
Tyrosol	leaves OMWW OMSW OMWW OMWW olive pomace	138.16	C_8_H_10_O_2_	450 ^b^ 1870 ^b^ 4 ^b^ 182 ^b^ 2043 ^b^ 162–3514 ^a,c^	Ladhari et al. [284] Poerschmann et al. [285] Benincasa et al. [283] Zhao et al. [282]
Hydroxytyrosol	leaves OMWW OMWW OMWW olive pomace	154.16	C_8_H_10_O_3_	130 ^b^ 4450 ^b^ 225 ^b^ 1481 ^b^ 1356–17,298 ^a,c^	Ladhari et al. [284] Poerschmann et al. [285] Benincasa et al. [283] Zhao et al. [282]
Flavonoids					
Luteolin	leaves OMWW OMSW olive pomace OMWW	286.24	C_15_H_10_O_6_	2970 ^b^ 1010 ^b^ 4 ^b^ 10–3515 ^b,c^ 62.38 ^b^	Ladhari et al. [284] Zhao et al. [282] Benincasa et al. [283]
Luteolin 7-*O*-glucoside	leaves OMWW olive pomace	448.37	C_21_H_20_O_11_	7620 ^b^ 150 ^b^ 42–4086 ^b,c^ 88.55 ^b^	Ladhari et al. [284] Zhao et al. [282] Benincasa et al. [283]
Luteolin 7-*O*-rutinoside		594.51	C_27_H_30_O_15_		
Luteolin 4′-*O*-glucoside	OMWW	448.37	C_21_H_20_O_11_	11.48 ^b^	Benincasa et al. [283]
Rutin	leaves OMWW deffated olives olive pomace	610.52	C_27_H_30_O_16_	110 ^b^ 110 ^b^ 3.3 ^a^ 770–11,048 ^b,c^ 48.52 ^b^	Ladhari et al. [284] Alu’datt et al. [281] Uribe et al. [286] Zhao et al. [282] Benincasa et al. [283]
Hesperidin	deffated olives	610.56	C_28_H_34_O_15_	7.4 ^a^	Alu’datt et al. [281]
Quercetin	leaves OMWW OMSW deffated olives	302.24	C_15_H_10_O_7_	4390 ^b^ 1060 ^b^ 37 ^b^ 5.7 ^a^	Ladhari et al. [284] Alu’datt et al. [281]
Apigenin		270.24	C_15_H_10_O_5_	7–469 ^b,c^	Benincasa et al. [283] Zhao et al. [282]
Apigenin 7-*O*-glucoside		432.38	C_21_H_20_O_10_	55–1345 ^b,c^	Zhao et al. [282]

* OMWW—olive mill wastewater, ** olive mill solid waste, ^a^ percentage of total phenolic content based on peak areas, ^b^ expressed in mg/g dry weight, ^c^ depending on the methods of extraction.

**Table 21:** Biological activity and potential applications of phytochemicals obtained from olive waste.

Material	Extract/Compound	Biological Activity/Application	References
olive leave	extract	- antioxidant, antimicrobial - antitumor activity - reduction of the risk of coronary heart disease	Taamalli et al. [288]
OMWW *	phenolic extract	- antioxidant activity - DPPH radical-scavenging activity	Kreatsouli et al. [291]
pressed olive cake	phenolic compounds	- superoxide anion scavenging - LDL oxidation - the protection of catalase against hypochlorous acid	Alu’datt et al. [281]
Olive oil mill waste	SFE extract and ethanol extract (hydroxytyrosol as the main compound)	- antioxidant activity - DPPH radical-scavenging activity - application as an antioxidant act against peroxidation of virgin olive and sunflower oils	Lafka et al. [292]
OMWW	polyphenolic fraction	- formulation of ophthalmic hydrogel containing a polyphenolic fraction	Di Mauro et al. [294]
dried olive mill wastewater	polyphenols	- application as ingredients in the food industry for obtaining functional and nutraceutical foods, as well as in the pharmaceutical industry	Benincasa et al. [297]
OMWW	polyphenol fraction	- antibacterial activities against *Staphylococcus aureus*, *Bacillus subtilis*, *Escherichia coli*, and *Pseudomonas aeruginosa*	Obied et al. [298]
		- fungicidal activities	Yangui et al. [299]
olive leaves and olive pomace	phenolic compounds	- ability as antimicrobial, antifungal, antitoxigenic to reduce aflatoxigenic fungi hazard and its aflatoxins - application as a manufacturing process, like, food supplement or preservatives	Abdel–Razek et al. [300]
olive leaves	IR extract	- antiradical activity - antioxidant activity - inhibition of the growth of *Aspergillus flavus* and production of aflatoxin B_1_ - inhibition of 20 strains of *Staphylococcus aureus*	Abi–Khattar et al. [302]
OMWW	hydroxytyrosol	cytoprotection of brain cell	Schaffer et al. [303]

* OMWW—olive mill wastewater.

## Data Availability

Not applicable.
